# Spore forming Actinobacterial diversity of Cholistan Desert Pakistan: Polyphasic taxonomy, antimicrobial potential and chemical profiling

**DOI:** 10.1186/s12866-019-1414-x

**Published:** 2019-02-22

**Authors:** Adeela Fatima, Usman Aftab, Khaled A. Shaaban, Jon S. Thorson, Imran Sajid

**Affiliations:** 1 Department of Microbiology and Molecular Genetics (MMG), University of the Punjab, Quaid-e-Azam Campus, Lahore, 54590 Pakistan; 2 Center for Pharmaceutical Research and Innovation, College of Pharmacy, University of Kentucky, Lexington, KY 40536 USA; 3 Department of Pharmaceutical Sciences, College of Pharmacy, University of Kentucky, Lexington, KY 40536 USA

**Keywords:** Actinobacterial diversity, Cholistan desert, MRSA, Polyphasic taxonomy, Scanning electron microscopy (SEM), 16S rRNA gene sequencing

## Abstract

**Background:**

Actinobacteria are famous for the production of unique secondary metabolites that help in controlling the continuously emerging drug resistance all over the globe. This study aimed at the investigation of an extreme environment the Cholistan desert, located in southern Punjab, Pakistan, for actinobacterial diversity and their activity against methicillin resistant *Staphylococcus aureus* (MRSA). The Cholistan desert is a sub-tropical and arid ecosystem with harsh environment, limited rainfall and low humidity. The 20 soil and sand samples were collected from different locations in the desert and the actinobacterial strains were selectively isolated. The isolated strains were identified using a polyphasic taxonomic approach including morphological, biochemical, physiological characterization, scanning electron microscopy (SEM) and by 16S rRNA gene sequencing.

**Results:**

A total of 110 desert actinobacterial strains were recovered, which were found to be belonging to 3 different families of the order *Actinomycetales*, including the family *Streptomycetaceae*, family *Pseudonocardiaceae* and the family *Micrococcaceae*. The most frequently isolated genus was *Streptomyces* along with the genera *Pseudonocardia* and *Arthrobacter*. The isolated strains exhibited promising antimicrobial activity against methicillin resistant *Staphylococcus aureus* (MRSA) with zone of inhibition in the range of 9–32 mm in antimicrobial screening assays. The chemical profiling by thin layer chromatography, HPLC-UV/Vis and LC-MS analysis depicted the presence of different structural classes of antibiotics.

**Conclusion:**

The study revealed that Cholistan desert harbors immense actinobacterial diversity and most of the strains produce structurally diverse bioactive secondary metabolites, which are a promising source of novel antimicrobial drug candidates.

**Electronic supplementary material:**

The online version of this article (10.1186/s12866-019-1414-x) contains supplementary material, which is available to authorized users.

## Background

**Fig. 1:**
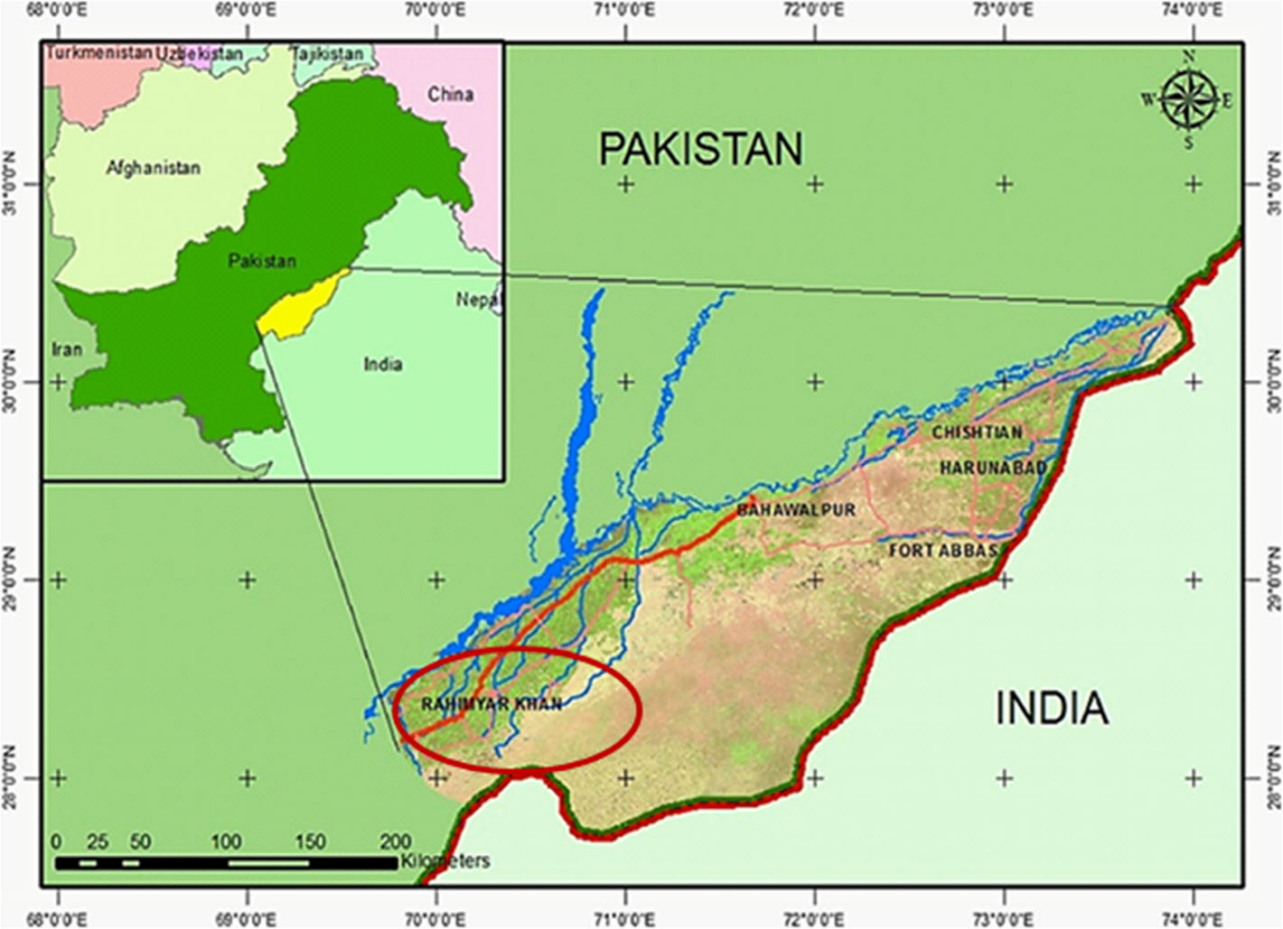
GIS Location map of Cholistan desert Pakistan, Islam et al. [1], (Map used with permission)

The actinomycetes thriving in harsh environments have the capability to endure the extreme temperature, drought conditions and to produce very unique antibacterial compounds. The desert soils are rich and untapped source of novel strains of these extraordinary species. There is a need to explore and screen these niches for the diversity of these prolific sources of antibiotics to combat the alarming situation of antimicrobial resistance. The Cholistan desert in Pakistan is a unique and unexplored ecological system covering the expanse of 26,300 km^2^ in the south of district Bahawalpur in the Punjab. It extends to the Thar desert in Sindh between longitudes 69°52′ to 73°24′ E and latitudes 28°42′ to 29°25′N (Fig. 1), present at an altitude of 89 m above sea level [1]. With reference to the climate, it is categorized as harsh, sub-tropical region with scarcity of precipitation and long dearth season, low relative humidity, with high rate of evaporation [2, 3]. The soil is very poor in organic matter and it may possess unique actinomycetes with unusual antibacterial activity. Although the actinomycetes are present in various environments like soil, fresh water, marines, in the plant tissues etc. but those present in unexplored habitats may have some unique characteristics to live in such habitats and to produce unique secondary metabolites [4]. These are abundantly present in soil of different types, but the arid habitats like deserts are now becoming the target ecosystems for their search because of the uniqueness of environmental conditions.

Because of the ease of access to antibiotics, the misuse of these drugs has been increased, as a result we are lacking in having a perfect antibiotic which can eradicate the pathogens without causing harmful side effects [5]. New antibiotics are required to fight against the counterfeiting pathogens that have developed resistance against existing antibiotics [6]. Methicillin resistant *Staphylococcus aureus* (MRSA) has been designated as one of the major hazardous pathogens associated with the development of antimicrobial resistance (AMR). The advent of AMR in *S. aureus* is widely recorded in Pakistan and around the globe and the species has proven particularly skilled at evolving resistance in the face of new antibiotic challenges. Therefore, the search for unrevealed species is critically needed to address this emerging problem and there is a recommendation to explore the untapped environments like deserts, caves *etc* [7].The exploration of new species of actinobacteria will likely give a chance to discover potentially new structural and beneficial secondary metabolites. The actinomycetes isolated from deserts have the capability to survive the harsh conditions of weather like high pH of the soil and salinity, and they possess unusual gene clusters for the production of unique and potent antibacterial compounds [8].

Different techniques are being used to identify these unique bacterial species and the compounds produced by them like microscopic analysis [9]. Microscopic studies especially scanning electron microscopic (SEM) examination of different species of actinomycetes provides useful information about their ultra-structures. The spores are considered to be the electron dense structures and the examination of their surfaces and arrangement in the form of chains and clusters has been studied by SEM. The spore arrangement is considered as an important criterion for the determination of the taxonomic position of actinomycetes in different groups. The use of SEM is increasing for the classification of actinomycetes because of its higher magnification and great focal strength [10]. Now, very high quality polycarbonate filters are available with different pore sizes for the collection of viruses and bacteria which are suitable for their surface observation using SEM [11].

A little information is present regarding the actinomycetes isolated from harsh and arid ecosystems, which are considered as the most bountiful environments for the discovery of new bacteria and compounds [8]. In our previous report [12], we reported the identification and antimicrobial activity of some selected strains from this ecosystem against methicillin resistant *Staphylococcus aureus* (MRSA), with major emphasis on the identification and validation of MRSA strains from clinical samples. This study reports a detailed account of the search and screening of the unique and extreme ecological niche; the Cholistan desert, Pakistan for the culture-able spore forming actinobacterial diversity, along with the in depth chemical profiling of the bioactive compounds produced by them. We believe that this ecosystem has not been studied for the detailed actinobacterial diversity, so this seems to be the first comprehensive report on culture-able spore forming actinobacterial diversity of the Cholistan desert Pakistan.

## Methods

### Sample collection

The 20 soil samples were collected in polythene bags from various sites in Cholistan desert Pakistan. The samples were obtained at least 1–2 km distance from each other. These samples were then processed first by applying different physical and chemical treatments (Heat treatment was given at 50–55 °C for 2–16 h and chemical treatment was given by mixing the soil sample with CaCO_3_ in the ratio 10:1 (*w*/w) followed by incubation at room temperature for 7–9 days in humid environment) by adopting the methods narrated by Hayakawa and Nonomura [13] for the selection of actinobacteria.

### Isolation of Actinobacteria

For the isolation of actinobacterial strains, serial dilution method was adopted for each sample. Two different isolation media were used according to the guidelines of International *Streptomyces* Project (ISP) for the isolation of variety of strains: glycerol-casein-KNO_3_ agar (Sigma) and actinomycete isolation agar (media recipes are included in additional file 1) were prepared and sterilized by autoclaving. To prevent or suppress the fungal growth, cycloheximide (20 mg/L) was added in the media after autoclaving. The serial dilutions of the samples were prepared and 50 μl of the dilutions 10^− 2^ and 10^− 3^ were spreaded on isolation media, followed by incubation at 28 °C for 7 to 15 days for both of the media. The prospective actinobacterial colonies were chosen from media plates and were streaked on the enriched medium i.e. Yeast extract-malt extract (GYM) agar (Sigma) [14] and then purified by repeated streaking on GYM agar. The pure cultures were preserved as glycerol stocks at − 80 °C in the microbial storage facility at the Department of Microbiology and Molecular Genetics (MMG), University of the Punjab (a redundant copy of the strains has also been stored in the collaborating laboratory at the Department of Pharmaceutical Sciences, College of Pharmacy, University of Kentucky). The strains were initially screened for the antimicrobial activity against MRSA by the agar plug method or the so called solid media bioassay [15] and active strains in whole collection were detected (Additional file 2; Table S1).

### Taxonomic characterization of the Actinobacterial strains

A polyphasic taxonomic approach was adopted for the identification of the isolated strains. All of the strains were studied for morphological, physiological and biochemical characteristics, along with the 16S rRNA gene sequencing of the 30 representative strains. The morphological characterization was performed with the pure and individual strains by following the methods of Bensultana et al. [16]. The features observed, included colony morphology, color of aerial and substrate mycelia and pigmentation. The strains were probed for biochemical features including the production of melanin, utilization of different sugars, organic acid formation, utilization of oxalate and organic acid, hydrolysis of esculin & urea.

For genetic characterization, the DNA of actinobacterial strains was extracted from mycelial part, using tissue genomic DNA extraction kit (FavorPrep™, Cat# FATGK001–1). The 16S rRNA gene sequence of these DNA samples were amplified by PCR amplification, using universal primers (27f: AGAGTTTGATCCTGGCTCAG) and (1522r: AAGGAGGTGATCCARCCGCA) [17]. The PCR reaction mixture included 5 μl of DNA sample, 2 μl of each primer with working concentration of 10 pmol and 25 μl of 2 X PCR master mixes (THERMO-scientific DreamTaq Green Master Mix). The reaction conditions were as follows: 95 °C for 1 min, 30 cycles of 95 °C for 30 s, annealing at 55 °C for 30 s, extension at 72 °C for 30 s followed by final extension for 5 min at 72 °C [18]. Amplified products were purified by employing the protocol described in gel purification kit (FavorPrep™, Cat# FAGPK001–1), then sequenced on an automated sequencer. The sequenced data obtained was BLAST analyzed at the NCBI [19] as well as at EzBioCloud database [20], for determining the similarity with type strains. The analyzed sequences of each strain were deposited to the NCBI GenBank to get the accession numbers. The evolutionary history was inferred by constructing phylogenetic tree by Neighbor-Joining method using 1000 replicates bootstrap value [21, 22]. The evolutionary distances were computed using the Maximum Composite Likelihood method [23] and are in the units of the number of base substitutions per site. Evolutionary analyses were conducted in MEGA6 [24].

### Scanning Electron microscopy (SEM)

The ultra-structure of mycelium and arrangement of spores of representative desert actinobacterial strains was observed by using scanning electron microscopy following the procedure described by Cavaletti et al. [25]. A small piece of GYM agar from the purified culture plate of actinobacteria was trimmed with a sterilized scalpel and fixed with a solution of cacodylate buffer having 5% formaldehyde and 2% glutaraldehyde (0.1 M cacodylate, 0.01 M CaCl_2_, 0.01 M MgCl_2_, 0.09 M sucrose; pH 6.9) for 3 h on ice and washed with the cacodylate buffer. Dehydration of the samples was done by using different grades of acetone (10, 30, 50, 70, 90, and 100% acetone) and each step was performed on ice block for 30 min. After that the samples were dried by using liquid CO_2_ and covered with an approximately 10 nm thick gold film by spattered coating. These samples were then observed under a range of magnifications (5.00 kx, 10.0 kx, 15.0 kx, 20.0 kx, 25.0 kx, 40.0 kx and 50.0 kx) with acceleration voltage 5.0 kV, 7.0 kV and 10.0 kV in VEGA3 (TESCAN) Scanning Electron Microscope by using SE (secondary electron) detector.

### Preparation of Methanolic extracts of desert Actinobacteria

The desert actinobacterial isolates were grown on lab scale as shaking flask cultures (250–500 ml GYM broth) and the crude extracts were obtained by solvent-solvent extraction using a separating funnel. The equal volumes of culture broth and ethyl acetate were mixed in the separating funnel and shaken for about 15 min, after which the mixture was allowed to settle down. After the two distinct layers appeared, the upper organic layer was retrieved carefully and the procedure was repeated thrice. The ethyl acetate was then evaporated on rotary vacuum evaporator (Heidolph Laborota 4000 efficient, Type# D-91126 Schwabach) and the extract was dissolved in analytical grade methanol.

### Determination of antibacterial activity against MRSA

The antimicrobial activity of desert actinobacterial strains was determined at two levels, initially all the isolated 110 strains were screened by agar plug method against one MRSA strain and active strains were selected. Later the active strains were further screened by well diffusion methods against the 10 MRSA strains. For this purpose ten strains of MRSA were collected from a diagnostic laboratory (Citi Lab, Lahore). These strains were confirmed as MRSA by performing different biochemical tests (catalase and DNase), through disc diffusion antibiotic assay using a panel of 10 antibiotics discs including methicillin (10 μg), oxacillin (1 μg), cefoxitin (30 μg), ampicillin (10 μg), amikacin (30 μg), amoxicillin (25 μg), cephalexin (30 μg), cefoperazone (75 μg), ciprofloxacin (5 μg) and piperacillin (100 μg) according to the CLSI standards [26]. The validation of these strains was also established genetically by *mec-A* gene and 16S rRNA gene sequencing [12] (GenBank Accession numbers: KU662352-KU662355, KR862284, KR862285, KR862287- KR862289, KR862291).

The discs of agar (5 mm) were cut from the pure culture plates of actinobacterial strains and placed on the MH (Muller Hinton agar) plates swabbed with MRSA isolate and zones of inhibition were recorded after overnight incubation [27]. The well diffusion method was performed by loading 60 μl of methanolic extracts of actinobacterial isolates in the wells (5 mm diameter) made in MH agar swabbed with MRSA isolate. The zones of inhibition were measured around the wells after overnight incubation [27].

### Chemical profiling of the Actinobacterial strains

The methanolic extracts of the bioactive actinobacterial strains were screened for their chemical profile by employing three chromatographic techniques: thin layer chromatography, high performance liquid chromatography (HPLC) and LC-MS.

### Thin layer chromatography

Crude extracts of actinobacterial strains were tested on TLC plates (TLC silica gel 60 F_254_, Merck, Germany) to assess the nature of secondary metabolites produced by them. Each of the extracts was superimposed onto two TLC plates by repeated spotting using a small capillary. The loaded plate was run in 10% methanol/dichloromethane solvent system and visualized in UV light (two wavelengths were used i.e., 254 nm and 366 nm) using a UV lamp (CAMAG). After the development, one plate was stained with anisaldehyde/H_2_SO_4_ spraying reagent (anisaldehyde, methanol, H_2_SO_4_, acetic acid) while the second plate was sprayed by Ehrlich’s reagent (HCl 37%, 4-dimethylamino benzaldehyde, methanol) in order to determine different colored bands of the compounds in the extracts.

### High performance liquid chromatography (HPLC-UV/Vis) analysis

HPLC-UV/Vis analysis of crude extracts of actinobacterial strains was carried out on clarity chromatography software in Sykum® S1122 delivery system. The separation of the components of secondary metabolites was carried out by using RPC18 column (ThermoHypersil-Keystone, 250 × 4.6 mm, particle size: 5 μm) which act as a stationary phase. The mobile phase was methanol and water (9:1) while the rate of flow was set at 1 ml/min. Various peaks were observed in the UV chromatograms of the samples which were compared later with the UV chromatographic data of secondary metabolites to interpret the type of compounds produced by the actinobacterial strains.

### High performance liquid chromatography- mass spectrometry (HPLC-MS) analysis

HPLC-MS analysis of the most active actinobacterial extracts was performed on the Agilent 6120 Quadrupole MSD mass spectrometer (Agilent Technologies, Santa Clara, CA, USA) equipped with an Agilent 1200 Series Quaternary LC system and an Eclipse XDB-C18 column (150 × 4.6 mm, 5 μm; solvent A: H_2_O/0.1% formic acid, solvent B: CH_3_CN/0.1% formic acid; flow rate: 0.4 ml min^− 1^; 0–4 min, 10% B; 4–22 min, 10–100% B; 22–27 min, 100% B; 27–29 min, 100–10% B; 29–35 min, 10% B). The molecular masses on each of the ion peak were calculated in all the extracts and the data was analysed by comparison with the already reported *Streptomyces* compounds in the AntiBase [28].

## Results

### Taxonomic characteristics of the selected Actinobacterial strains

A total of 110 actinobacterial strains were recovered from the desert soil and sand samples. Most of the strains produced hard, embedded and rough textured colonies with different sizes and regular or irregular margins. A variety of colors of aerial mycelia were observed in different strains including white, yellowish white, yellow, purple, orange and pink *etc*, similarly the substrate mycelia displayed the colors including white, yellowish, brown, light pink, purple and orange *etc* (Table 1, Additional file 3: Table S2, Additional file 4: Figure S1. The diffused pigments were observed only in 3 strains including, AFD1 (brown), AFD15 (light brown) and in AFD26 (dark yellow) (Table 1, Additional file 3: Table S2, Additional file 4; Figure S1).

**Table 1:** Morphological characteristics of the selected Cholistan desert actinobacterial strains observed on GYM agar after cultivation at 28 °C for one week

Strains	^a^Isolation codes	Growth Pattern	Color of Mycelium		Diffused pigment	Colony characteristics		
			Substrate Mycelium	Aerial Mycelium		Consistency	Size mm	Shape
AFD1	AD3	Well grown	White	Grayish white	Brown	Hard & embedded	3	Round
AFD2	AD6	Well grown	Light yellow	Yellowish white	No pigment	Hard & embedded	3	Round
AFD3	AD10	Well grown	Grayish white	White	No pigment	Hard & embedded	4	Round
AFD4	AD15	Well grown	Light pink	Whitish yellow	No pigment	Soft with spores	2	Round
AFD5	AD20	Moderate growth	White	Yellowish white	No pigment	Hard & embedded	2	Round
AFD6	AD21	Well grown	Brown	Yellow	No pigment	Hard & embedded	3	Round
AFD7	AD23	Well grown	Yellowish white	White	No pigment	Hard & embedded	2	Round
AFD8	AD29	Well grown	White	Yellowish white	No pigment	Hard & embedded	3	Round
AFD9	AD34	Well grown	Light pink	White	No pigment	Soft with spores	2	Round
AFD10	AD37	Well grown	Purple pink	Purple	No pigment	Hard & embedded	2	Round
AFD11	AD38	Moderate growth	Yellowish white	White	No pigment	Hard with less spores	3	Round
AFD12	AD42	Well grown	White	Whitish grey	No pigment	Hard & embedded	3	Round
AFD13	AD48	Well grown	Light orange	Yellowish orange	No pigment	Hard & embedded	3	Round
AFD14	AD54	Well grown	Purple pink	Purple	No pigment	Soft with spores	2	Round
AFD15	AD57	Well grown	Brown	Light brown	Light brown	Hard & embedded	3	Round
AFD16	AD59	Well grown	White	Greenish brown	Brown	Hard & embedded	2	Round
AFD17	AD61	Well grown	Yellowish white	White	No pigment	Hard & embedded	3	Round
AFD18	AD63	Well grown	Yellowish white	Yellow	No pigment	Hard & embedded	3	Round
AFD19	AD68	Well grown	Purple pink	Purple	No pigment	Hard & embedded	2	Round
AFD20	AD69	Well grown	White	Grey white	No pigment	Hard & embedded	2	Round
AFD21	AD73	Well grown	Orange yellow	Orange	No pigment	Hard & embedded	3	Round
AFD22	AD76	Well grown	Orange white	Orange	No pigment	Hard & embedded	2	Round
AFD23	AD80	Well grown	Yellow white	Yellow	No pigment	Soft with spores	2	Round
AFD24	AD81	Well grown	White	Yellowish white	No pigment	Hard & embedded	2	Flower shaped
AFD25	AD85	Moderate growth	Purple	Pink	No pigment	Hard & embedded	3	Round
AFD26	AD89	Well grown	Meroon or dark brown	Yellowish white	Dark Yellow	Hard & embedded	2	Round
AFD27	AD96	Well grown	Brown	White brown	Yellow	Hard & embedded	3	Round
AFD28	AD100	Well grown	Yellowish white	White	No pigment	Hard & embedded	3	Round
AFD29	AD104	Well grown	White	Whitish grey	No pigment	Hard & embedded	2	Round
AFD30	AD107	Moderate growth	White	Yellowish white	No pigment	Hard & embedded	2	Round

^**a**^the strains were designated by specific number codes based on the sequence of isolation at the time of selection from a sample

In case of the testing of different sugars as sole source of carbon, the glucose and mannose were utilized by all of the strains except the strain, AFD23. Similarly fructose was used as carbon source by most of the strains, except four strains including AFD6, AFD14, AFD20 and AFD26 (Table 2). Sixteen of the strains exhibited the tyrosinase activity and production of melanin. Most of the strains showed hydrolysis of urea and esculin, utilization of organic acids and oxalate etc. Similarly most of the strains were found positive in case of organic acid formation and lecithovitellin reaction (Table 2, Additional file 3: Table S2).

**Table 2:** Biochemical and physiological characteristics of Cholistan desert actinobacterial strains

Strains	Hydrolysis of		Utilization of organic acids		Oxalate utilization	Organic acid formation	Lecithovitellin reaction	Sugar utilization profile								
	Urea	Esculin	C	M				MP	DG	DF	LA	DM	S	I	DGL	M
AFD1	**+**	**+**	**+**	**+**	**+**	**+**	**+**	**+**	**+**	**+**	**+**	**+**	**+**	**+**	**+**	**+**
AFD2	**+**	**+**	**+**	**+**	**+**	**+**	**–**	**+**	**+**	**+**	**+**	**+**	**+**	**+**	**+**	**+**
AFD3	**+**	**–**	**+**	**–**	**–**	**+**	**+**	**+**	**+**	**+**	**+**	**–**	**–**	**+**	**+**	**+**
AFD4	**+**	**–**	**–**	**–**	**–**	**–**	**–**	**–**	**+**	**+**	**–**	**–**	**+**	**–**	**–**	**+**
AFD5	**+**	**+**	**+**	**+**	**+**	**–**	**+**	**–**	**+**	**+**	**+**	**+**	**–**	**+**	**–**	**+**
AFD6	**–**	**+**	**–**	**+**	**–**	**+**	**+**	**+**	**+**	**–**	**+**	**+**	**–**	**–**	**+**	**+**
AFD7	**+**	**–**	**–**	**–**	**–**	**–**	**–**	**+**	**+**	**+**	**–**	**+**	**–**	**–**	**–**	**+**
AFD8	**+**	**+**	**+**	**–**	**+**	**–**	**+**	**–**	**+**	**+**	**+**	**–**	**–**	**+**	**–**	**+**
AFD9	**+**	**+**	**+**	**+**	**+**	**+**	**–**	**+**	**+**	**+**	**+**	**+**	**+**	**+**	**+**	**+**
AFD10	**+**	**+**	**+**	**+**	**+**	**+**	**+**	**–**	**+**	**+**	**+**	**+**	**+**	**+**	**+**	**+**
AFD11	**+**	**–**	**–**	**–**	**–**	**+**	**–**	**–**	**+**	**+**	**–**	**+**	**–**	**+**	**+**	**+**
AFD12	**+**	**–**	**+**	**–**	**–**	**–**	**–**	**+**	**+**	**+**	**–**	**+**	**–**	**+**	**–**	**+**
AFD13	**+**	**+**	**–**	**+**	**+**	**–**	**+**	**+**	**+**	**+**	**+**	**–**	**–**	**–**	**–**	**+**
AFD14	**–**	**+**	**–**	**+**	**+**	**–**	**+**	**–**	**+**	**–**	**+**	**–**	**–**	**–**	**–**	**+**
AFD15	**+**	**+**	**–**	**+**	**+**	**+**	**+**	**+**	**+**	**+**	**+**	**–**	**–**	**–**	**+**	**+**
AFD16	**+**	**–**	**–**	**–**	**–**	**–**	**–**	**–**	**+**	**+**	**–**	**–**	**–**	**–**	**–**	**+**
AFD17	**+**	**–**	**–**	**–**	**–**	**–**	**–**	**–**	**+**	**+**	**–**	**–**	**–**	**–**	**–**	**+**
AFD18	**+**	**+**	**–**	**+**	**+**	**+**	**+**	**+**	**+**	**+**	**+**	**–**	**–**	**–**	**+**	**+**
AFD19	**+**	**+**	**–**	**+**	**+**	**–**	**+**	**–**	**+**	**+**	**+**	**–**	**+**	**–**	**–**	**+**
AFD20	**–**	**+**	**–**	**+**	**+**	**–**	**+**	**+**	**+**	**–**	**+**	**–**	**–**	**–**	**–**	**+**
AFD21	**+**	**–**	**+**	**–**	**–**	**+**	**–**	**+**	**+**	**+**	**–**	**+**	**–**	**+**	**+**	**+**
AFD22	**+**	**+**	**+**	**+**	**+**	**+**	**+**	**+**	**+**	**+**	**+**	**+**	**+**	**+**	**+**	**+**
AFD23	**+**	**–**	**+**	**–**	**–**	**–**	**–**	**+**	**+**	**+**	**–**	**+**	**–**	**+**	**–**	**–**
AFD24	**+**	**+**	**+**	**+**	**+**	**+**	**+**	**–**	**+**	**+**	**+**	**+**	**+**	**+**	**+**	**+**
AFD25	**+**	**+**	**+**	**+**	**+**	**+**	**+**	**–**	**+**	**+**	**+**	**+**	**–**	**+**	**+**	**+**
AFD26	**–**	**+**	**+**	**+**	**+**	**+**	**+**	**–**	**+**	**–**	**+**	**+**	**–**	**+**	**+**	**+**
AFD27	**+**	**–**	**–**	**–**	**–**	**+**	**–**	**+**	**+**	**+**	**–**	**+**	**–**	**–**	**+**	**+**
AFD28	**+**	**–**	**–**	**–**	**–**	**+**	**–**	**–**	**+**	**+**	**–**	**+**	**–**	**–**	**+**	**+**
AFD29	**+**	**–**	**–**	**–**	**–**	**–**	**–**	**+**	**+**	**+**	**–**	**–**	**–**	**–**	**–**	**+**
AFD30	**+**	**+**	**–**	**+**	**+**	**+**	**+**	**–**	**+**	**+**	**+**	**–**	**–**	**–**	**+**	**+**

Key: *C* citrate, *M* Melonate, *MP* melanin production, *DG* D-glucose, *DF* D-fructose, *LA* L-arabinose, *DM* D-mannitol, *S* Sucrose, *I* Inositol, *DGL* D-galactose, *M* Mannose, (+) = positive result, (−) = negative result

**Fig. 2:**
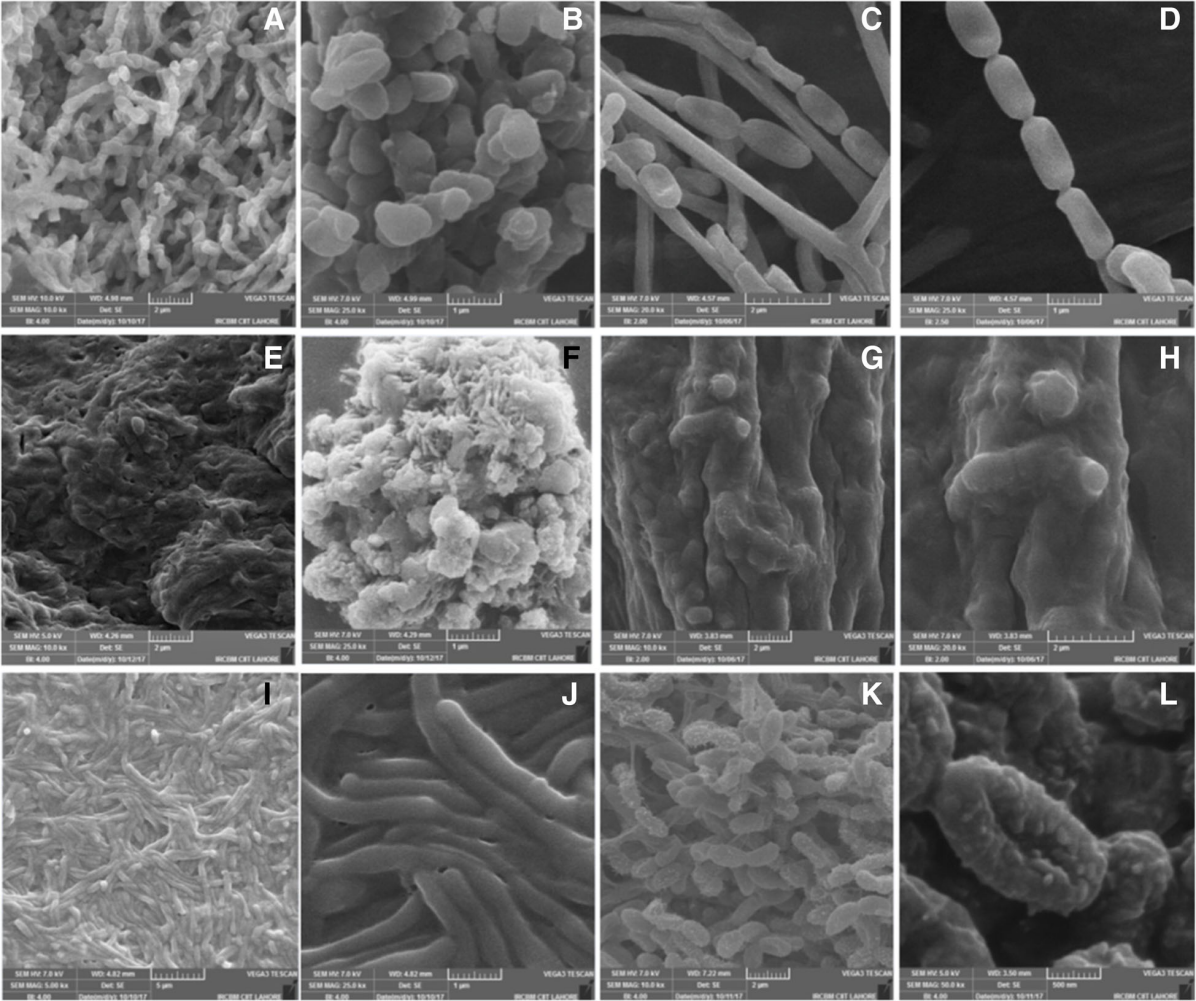
Scanning electron microscope images of Cholistan desert actinobacteria under different magnifications: Strain AFD1 (**a**) 10.0 kx, (**b**) 25.0 kx; Strain AFD7 (**c**) 20.0 kx, (**d**) 25.0 kx; Strain AFD8 (**e**) 10.0 kx, (**f**) 25.0 kx; Strain AFD16 (**g**) 10.0 kx, (**h**) 20.0 kx; Strain AFD19 (**i**) 5.0 kx (**j**) 40.0 kx; Strain AFD26 (**k**) 10.0 kx (**l**) 50.0 kx

In scanning electron microscopy using VEGA3 (TESCAN), different magnifications (5.0 kx, 10.0 kx, 20.0 kx, 25.0 kx, 40.0 kx, and 50.0 kx) revealed ultra-fine structure of the cells and arrangements of spores of actinobacterial strains, the best results were obtained by using 40.0 kx magnification (Fig. 2). As it can be seen in Fig. 2, the arrangement of spores in the form of chains is clear for all the isolates. In case of the strain AFD1, best results were obtained at higher magnification i.e. 25 kx. The spores are grouped in the form of chains with lateral depressions (Fig. 2a and b). In case of the strain AFD7 the spores can be seen entangled with each other to form the aerial mycelium on the agar surface. At higher magnification of the SEM (25 kx) the typical arrangement of the spores i.e. *Retinaculiaperti* [29] of this strain is clear (Fig. 2c and d). Some of the strains have unique pattern of spores arrangement as in case of strain AFD8 (Fig. 2e), showing a sheath of cells while in the Fig. 2f the spores are emerging from the cells like spines. Another unique arrangement was shown by the strain AFD16 (Fig. 2g, h) in which the spores are entangled to form an interlocking pattern. In case of the strain AFD19 long and mature spores can be seen as 10–50 spores per spore chain (Fig. 2i, j). In some strains, spiny surfaced spores can be seen with central depressions like in the case of strain AFD26 (Fig. 2k, l). The morphological (colony characteristics), microscopic (SEM), biochemical and physiological characteristics suggested that most of the isolated strains are the members of genus *Streptomyces* on comparison of the features with those of reported in the literature [29, 30].

The 16S rRNA gene sequences and BLAST analysis at EzBioCloud database [20] of the 29 representative strains showed %similarity with different actinobacterial type strains. In 25 of the cases %similarity in the range of 99 to 100% was observed with different species of the genus *Streptomyces* (Table 3). However 4 strains exhibited %similarity between 98 to 99%, such as strain AFD6 exhibited 98.68% similarity with the type strain *Streptomyces fradiae* (MIFZ01000280), the strains AFD23 and AFD26 exhibited 98.27 and 98.94% respectively, the %similarity with the type strain *Streptomyces albaduncus* (AY999757), the strain AFD29 showed 98.97% similarity with type strain *Streptomyces europaeiscabiei* (NR042790). While 1 strain AFD13 exhibited 97.02% similarity with the type strain *Streptomyces roseofulvus* (AB184327) (Table 3). Two of the strains exhibited %similarity with the type strains of genera other than *Streptomyces*, including the strain AFD18 showed 99.65% similarity with the type strain *Saccharothrix xinjiangensis* (AB381939), while the strain AFD22 showed 99.89% similarity with the type strain *Arthrobacter ANPE_s* (ANPE02000028) (Table 3).

**Table 3:** GenBank accession numbers of the selected actinobacterial strains and their similarities (percentage) with previously reported strains

Actino bacterial strains	Sequences submitted	GenBank accession numbers	Closely related taxa	Percentage similarity
AFD1	657 bp	MH552995	*Streptomyces setonii* (MUNB01000146)	100%
AFD2	992 bp	KX768011	*Streptomyces pseudogriseolus* (MUNG01000290)	99.8%
AFD3	978 bp	KX768009	*Streptomyces canarius* (AB184396)	99.69%
AFD4	877 bp	MH553082	*Streptomyces sedi* (EU925562)	100%
AFD5	868 bp	MH595998	*Streptomyces phaeofaciens* (AB184360)	99.98%
AFD6	1059 bp	KX131166	*Streptomyces fradiae* (MIFZ01000280)	98.68%
AFD7	190 bp	MG271834	*Streptomyces fradiae* (MIFZ01000280)	99.47%
AFD8	948 bp	MH553090	*Streptomyces stramineus* (AB184720)	100%
AFD9	1006 bp	KX768010	*Streptomyces iakyrus* (JNXI01000062)	99.90%
AFD10	349 bp	KX768012	*Streptomyces puniceus* (AB184163)	99.71%
AFD11	520 bp	MH553091	*Streptomyces artemisiae* (EU200685)	100%
AFD12	794 bp	KX131167	*Streptomyces werraensis* (AB184381)	99.62%
AFD13	711 bp	KX768013	*Streptomyces roseofulvus* (AB184327)	97.02%
AFD14	319 bp	KX768014	*Streptomyces puniceus* (AB184163)	100%
AFD15	1017 bp	MH607120	*Streptomyces griseoplanus* (NR118415)	99%
AFD16	738 bp	KX816586	*Streptomyces pratensis* (JQ806215)	100%
AFD17	891 bp	KX816590	*Streptomyces badius* (AY999783)	99.89%
AFD18	858 bp	KX094938	*Saccharothrix xinjiangensis* (AB381939)	99.65%
AFD19	742 bp	KX816592	*Streptomyces globisporus* (AB184203)	100%
AFD20	1011 bp	KX816587	*Streptomyces puniceus* (AB184163)	99.90%
AFD21	972 bp	KX816588	*Streptomyces alboflavus* (JNXT01000131)	99.90%
AFD22	891 bp	KX816589	*ANPE_s* (ANPE02000028)	99.89%
AFD23	1039 bp	KX131165	*Streptomyces albaduncus* (AY999757)	98.27%
AFD24	408 bp	KX816591	*Streptomyces pratensis* (JQ806215)	100%
AFD25	787 bp	KX131168	*Streptomyces werraensis* (AB184381)	99.49%
AFD26	947 bp	KX131169	*Streptomyces albaduncus* (AY999757)	98.94%
AFD28	733 bp	MH553095	*Streptomyces artemisiae* (EU200685)	99.86%
AFD29	868 bp	MH595999	*Streptomyces europaeiscabiei* (NR042790)	98.97%
AFD30	901 bp	MH628040	*Streptomyces lavendulae* (JOEW01000098)	99.55%

**Fig. 3:**
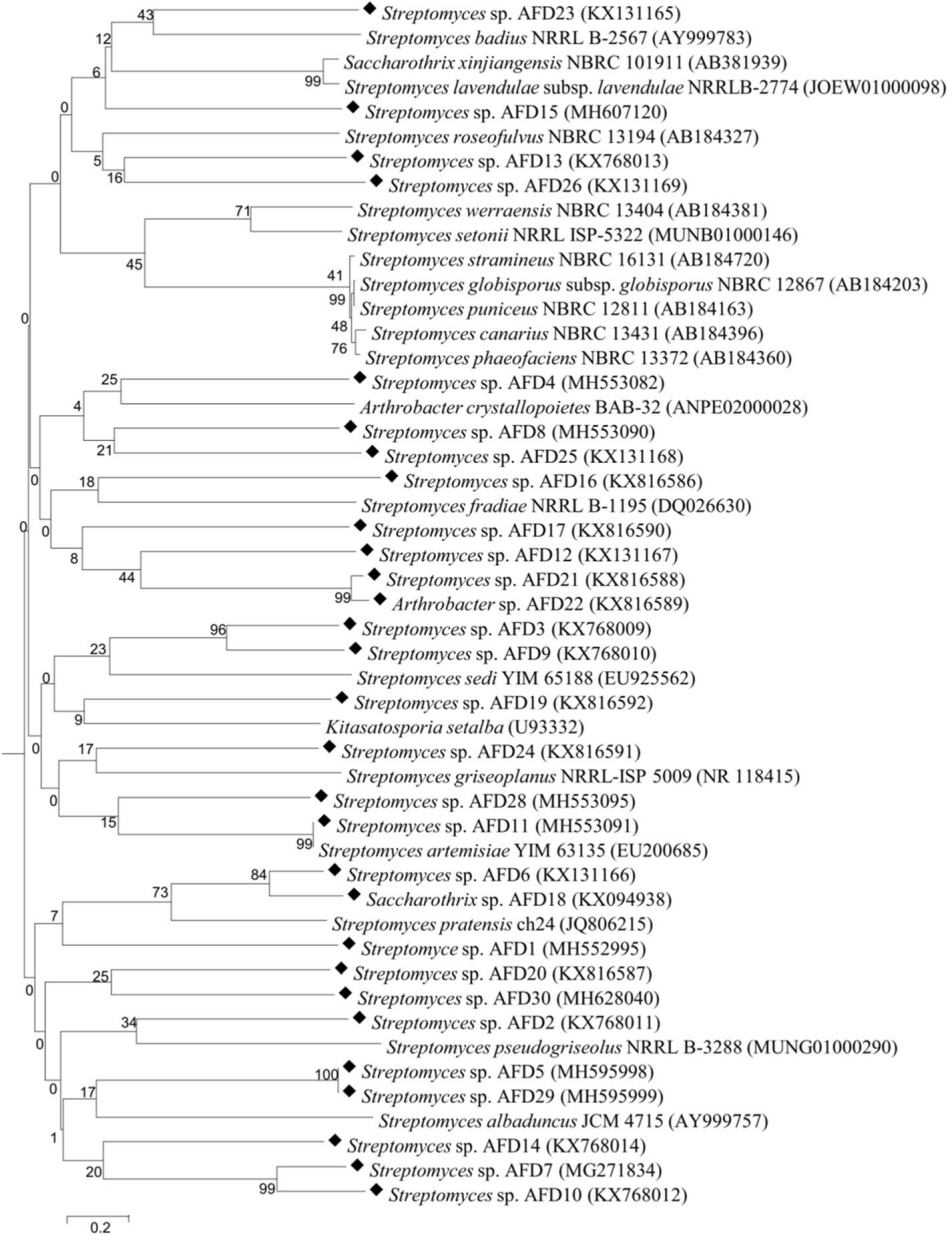
Molecular Phylogenetic analysis by Neighbor Joining method. The tree showing the interrelationships of isolated actinobacterial strains along with their closely related taxa belonging to *Streptomyces*, *Saccharothrix* and *Arthrobacter* inferred from 16S rRNA gene sequence. Data with gaps were removed after alignment by CLUSTAL W. The rooted tree was constructed using Neighbor Joining method. The analysis involved 49 nucleotide sequences. Codon positions included were 1st + 2nd + 3rd + Noncoding. There were a total of 254 positions in the final dataset. Evolutionary analyses were conducted in MEGA6

The neighbor joining phylogenetic tree constructed based on the 16S rRNA gene sequence data of the 29 strains, shows the phylogenetic relation of the strain with each other. The optimal tree with the sum of branch length = 32.44194265 is shown in Fig. 3. The tree is drawn to scale, with branch lengths in the same units as those of the evolutionary distances used to infer the phylogenetic tree. The evolutionary distances were computed using the Maximum Composite Likelihood method [23] and are in the units of the number of base substitutions per site. The analysis involved 49 nucleotide sequences, for which the NCBI GenBank accession numbers are given in parenthesis. Codon positions included were 1st + 2nd + 3rd + Noncoding. All positions containing gaps and missing data were eliminated. There were a total of 254 positions in the final dataset. Evolutionary analyses were conducted in MEGA6 [24]. The numbers at the nodes indicate the levels of bootstrap support based on 1000 replicates. The isolated strains showed phylogenetic relationships with each other and clustered in four major clads, showing various *Streptomyces* species with common ancestry. The strains made pairwise operational taxonomic units (OTUs) at species level with each other and with the closest match or type strains found by BLAST analysis via EzTaxon server (Fig. 3).

### Antibacterial activity of the selected Actinobacteria against MRSA

The actinobacterial strains displayed prolific antibacterial activity against MRSA isolates. In initial screening by agar plug method among the 110 isolated strains 30 strains showed growth inhibition of the MRSA test strain (Additional file 2: Table S1). The 30 active strains were further investigated for their inhibitory activity by well diffusion assay against a panel of 10 MRSA strains. In this secondary screening the most active strain was *Streptomyces pseudogriseolus* AFD2 which exhibited the maximum zone of inhibition up to 32 mm against the MRSA strain A11. The strain AFD4 displayed maximum zone of inhibition (18 mm) against MRSA strain A5. In the case of strain AFD7, maximum zone of inhibition was recorded to be 24 mm. The strain AFD8 exhibited maximum zone of inhibition against MRSA strain A1 and A5 i.e., 17 mm. The strain AFD9 showed maximum zone of inhibition 21 mm against MRSA strain A2. The strain AFD10 also exhibited very potent antibacterial activity with zone of inhibition of 20 mm against MRSA strains A2, A6 and A14. The strain AFD14 showed maximum zone of inhibition i.e., 23 mm against MRSA strain A7. The strain AFD15 exhibited maximum zone of inhibition against MRSA strain A9 i.e., 21 mm. Among other desert actinobacterial strains, another highly active strain was AFD16; it exhibited zone of inhibition up to 20 mm against MRSA strains A1, A5, A6 and A9. In the case of strain AFD20 maximum zone of inhibition recorded was 27 mm against MRSA strain A8 while in case of strain AFD21 maximum zone of inhibition recorded was 19 mm against MRSA strains A1, A5, A6 and A9. The strain AFD22 showed 26 mm zone of inhibition against MRSA isolates A1 and A14 (Table 4, Additional file 5: Figures S2A, S2B, S2C and S2D).

**Table 4:** Antibacterial activity of the selected actinobacterial strains against MRSA

Actinobacterial strains	Zone of inhibition (mm) against various MRSA strains									
	A1	A2	A5	A6	A7	A8	A9	A11	A12	A14
AFD1	19	14	18	12	15	14	15	16	13	12
AFD2	19	21	17	24	22	24	28	32	25	24
AFD3	11	12	19	11	15	14	12	14	11	19
AFD4	10	12	18	13	17	14	10	9	16	15
AFD5	11	13	15	12	13	15	12	–	12	10
AFD6	10	12	17	13	16	14	11	9	16	15
AFD7	24	22	23	24	25	24	21	20	20	21
AFD8	17	15	17	15	16	16	14	15	12	12
AFD9	18	21	17	18	13	15	15	16	13	14
AFD10	19	20	18	20	17	18	19	18	19	20
AFD11	10	12	13	12	–	13	14	–	11	13
AFD12	15	14	15	13	11	12	15	14	13	14
AFD13	14	14	15	11	10	12	11	12	12	14
AFD14	12	13	–	18	23	16	20	10	–	10
AFD15	11	16	11	11	13	15	21	19	19	13
AFD16	20	19	20	20	19	18	20	17	16	19
AFD17	12	13	13	–	–	10	–	–	10	12
AFD18	14	14	15	16	14	13	18	14	18	16
AFD19	10	11	12	13	12	10	13	10	11	12
AFD20	26	26	18	21	20	27	21	22	24	25
AFD21	19	17	19	19	18	16	19	18	16	13
AFD22	26	20	21	20	21	22	18	20	23	26
AFD23	14	13	12	16	16	14	12	14	10	15
AFD24	17	16	17	14	13	17	15	16	16	17
AFD25	10	14	11	16	–	15	17	10	16	12
AFD26	12	13	11	10	15	14	12	14	12	13
AFD27	9	9	10	10	–	13	11	10	10	14
AFD28	17	14	16	24	13	11	–	12	11	12
AFD29	13	17	14	14	12	14	11	11	10	10
AFD30	11	13	11	10	–	12	10	–	11	10

### Chemical profiling of the Methanolic extracts of Cholistan Desert Actinobacteria

In TLC analysis, the UV visible bands were observed at 254 nm and 366 nm wave length. The methanolic extracts of the strains AFD7, AFD9, AFD10, AFD20, AFD22, AFD24 and AFD26 developed some distinct bands when viewing in UV light (Additional file 5: Figure S2E, S2F). Different colored bands were observed in the extracts of the strains AFD9, AFD10, AFD20, AFD22 and AFD24 after treatment with anisaldehyde reagent. The spots of different colors like violet, blue, green, yellow and orange were seen after heating which expressed the presence of different structural classes of secondary metabolites like quinolones and actinomycins *etc* [31].

The HPLC-UV analysis of the crude extracts of the active strains exhibited varied peaks. For example, the crude extract of the actinobacterial strain AFD2 produced 3 prominent and many minor peaks, the highest was observed at 9.323 min, then at 2.876 min and 4.144 min (Additional file 6: Figure S3). The crude extract of another active strain AFD7 showed 6 peaks, the major was at retention time 4.113 min (Additional file 7: Figure S7). Besides these the crude extracts of other strains like AFD4, AFD8, AFD10, AFD12 and AFD26 also exhibited variety of peaks at different retention times (*t*_*R*_) with varied peak areas (Additional files 8: Figure S5, S9, S11, S13, S15 and S17, Additional files 9, 10, 11, 12 and 13).

**Fig. 4:**
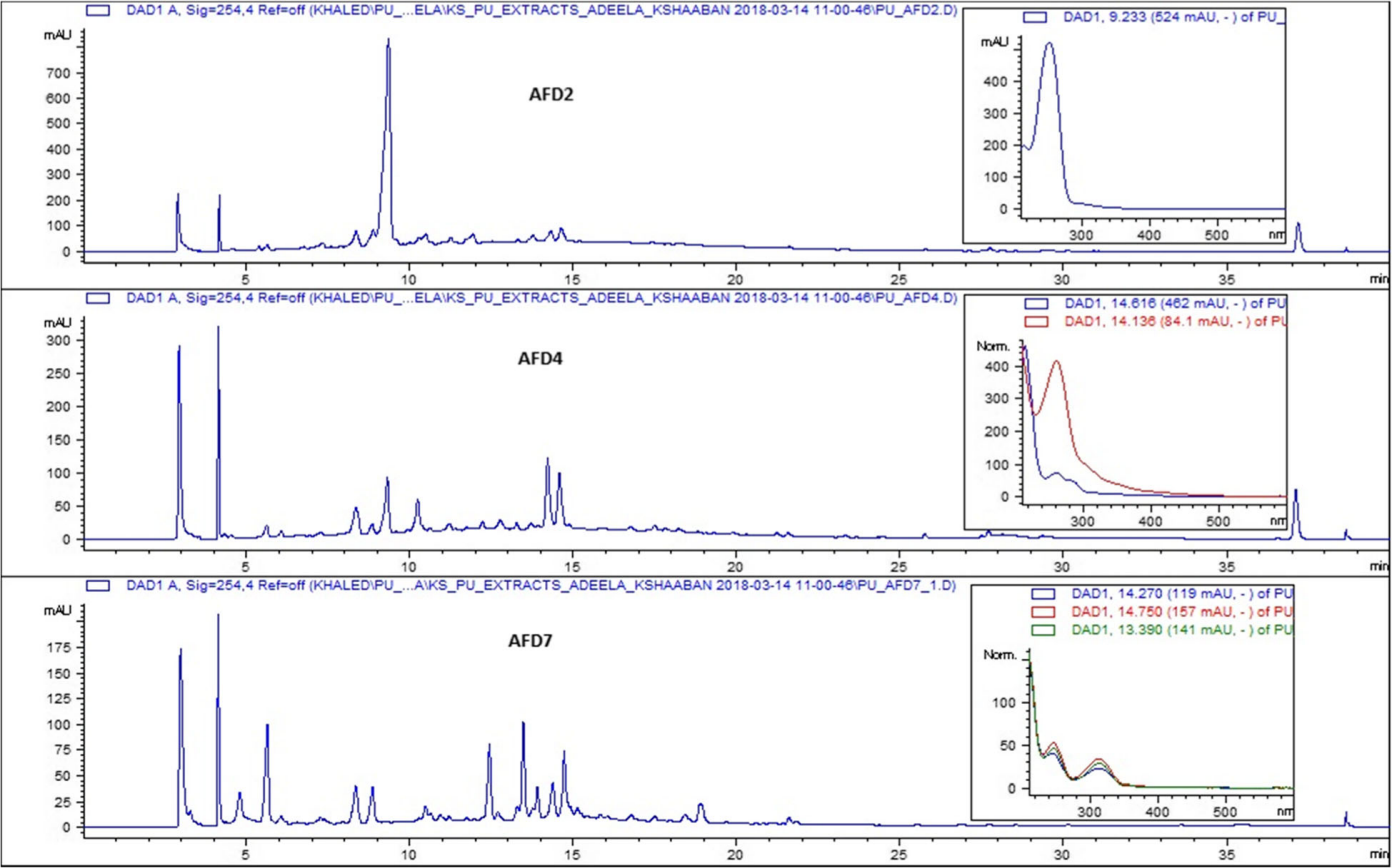
HPLC/UV analyses of the crude extracts produced by the actinobacterial strains AFD2, AFD4 and AFD7 (Detection wavelength 254 nm). For the full-expended versions of this figure, see Supplementary Information, Additional files 6, 8 and 7 (Figure S3, S5 and S7)

The LC-MS analysis of the extracts of most active actinobacterial strains displayed an exciting diversity of the components with antibacterial activity. As shown in Table 5, the total ion chromatogram of strain AFD2 showed various peaks at different retention times (*t*_*R*_) the most prominent of which was observed at *t*_*R*_ 6.38 min and the molecular mass calculated for this component was 112 Da. The comparison of the chemical profile of this component with the compounds in AntiBase gave the closest match with Emimycin, 3, 5-Dimethyl-3-oxol-2-on, and Uracil. Similarly the components at *t*_*R*_ 11.03 and 12.57 min in this extract gave closest match with various other compounds in AntiBase (Table 5, Fig. 4 and Additional files 14: Figure S4 and S19, Additional file 15).The total ion chromatogram of strain AFD4 showed varied peaks at different retention times (*t*_*R*_), such as a peak at 12.60 min with molecular mass 136 Da. The chemical profile of this component was then compared with the compounds in database and found the closest match with Pyrrole-3-yl-2-propenamide, Octalactin C and Anthranilamide. Likewise the components at *t*_*R*_ 10.23 and 12.22 min also showed close matches with the various compounds in the AntiBase (Table 5, Fig. 4 and Additional files 16: Figure S6, S19, Additional file 15). The total ion chromatogram of the strain AFD7 showed peaks at different retention times such as at *t*_*R*_ 3.83 min, molecular mass for which was calculated as 152 Da. The chemical profile of this component was compared with the compounds in AntiBase and the closest matches were observed with Xanthine, Guanazoloand Orsellin-aldehyde. The other components in this extract at other *t*_*R*_ 4.50 min, 5.77 min, 7.10 min, 10.24 min and 11.07 min were also compared in the same manner with the compounds in database and the prospective compounds were suggested (Table 5, Fig. 4 and Additional file 17: Figure S8, S19, Additional file 15).

**Table 5:** HPLC-MS analyses of the active desert actinobacterial strain

Strains	Thin Layer Chromatography (TLC) Profile			HPLC-Retention time (*t*_*R*_/min)	(+) and (−)-ESI-MS: (*m/z*)	Molecular Weights	Activity against MRSA	Suggested *Streptomyces* metabolites from *AntiBase* 2017 (Additional files 15 and 20)
	UV Visualization		Staining with Anisaldehyde/H_2_SO_4_					
AFD2	254 nm	4 bands	2green, 1purple, 2yellow	6.38 11.03 12.57	113 [M + H]^+^, 111[M-H]^−^ 208 [M + H]^+^, 206 [M-H]^−^ 137 [M + H]^+^, 135 [M-H]^−^	112 207 136	17 mm zone of inhibition against MRSA A5;32 mm zone of inhibition against MRSA A11	MW = 112: Emimycin, 3,5-Dimethyl-3-oxol-2-on, Uracil, Enimycin. MW = 207: Triacsin C, Triacsin D, Streptazolin, 2-(2-Hydroxypropionyl)acetanilide, Aeruginol, 7-Deoxyechinosporin. MW = 136: Pyrrole-3-yl-2-propenamide, Anthranilamide, Octalactin C.
	366 nm	2 bands						
AFD4	254 nm	3 bands	2 purple bands, 1 light blue band, 1 yellow band, 1 green band	10.23 12.22 12.60	211 [M + H]^+^, 209 [M-H]^−^ 146 [M + H]^+^ 137 [M + H]^+^, 135 [M-H]^−^	210 145 136	9 mm zone of inhibition against MRSA A11;18 mm zone of inhibition against MRSA A5	MW = 210: 1-Methoxyphenazine, 1-Acetyl-β-carboline MW = 146: Indole-3-carbaldehyde MW = 136: Pyrrole-3-yl-2-propenamide, Anthranilamide, Octalactin C.
	366 nm	2 bands						
AFD7	254 nm	4 bands	2 green bands, 2 yellow bands, 1 blue band	3.83 4.50 5.77 7.10 10.24 11.07 11.38 11.91 13.16 15.05 16.72	153 [M + H]^+^, 151 [M-H]^−^ 202 [M + H]^+^ 261 [M + H]^+^, 259 [M-H]^−^ 115 [M + H]^+^, 113 [M-H]^−^ 211 [M + H]^+^ 145 [M + H]^+^, 143 [M-H]^−^ 424 [M + H]^+^, 422 [M-H]^−^ 407 [M + H]^+^, 405 [M-H]^−^ 494 [M + H]^+^ 231 [M + H]^+^, 229 [M-H]^−^ 390 [M + H]^+^, 388 [M-H]^−^ 223 [M + H]^+^, 245 [M + Na]^+^	152 201 260 114 210 144 423 406 493 230 389 222	20 mm zone of inhibition against MRSA A11; 25 mm zone of inhibition against MRSA A7	MW = 152: Guanazolo, Orsellin-aldehyde, MY3–469, Xanthine. MW = 260: Cyclo(L-Tyr-L-Pro), VibrindoleA MW = 114: 4-Hydroxy-5-methyl-furan-3-one MW = 210: 1-Methoxyphenazine, 1-Acetyl-β-carboline MW = 144: Pentenomycin I, 4-Epipentenomycin I MW = 423: Fibrostatin B, A9145 A, 6-Amino-9 [(aminocarbonyl)amino]-1-(6-amino-9H-purin-9-yl)-1,5,6,7,8,9- hexadeoxy-decafuranuronamide MW = 389: Hatomamicin, SF-2809-I
	366 nm	4 bands						
AFD8	254 nm	4 bands	4 blue bands	9.07 13.42 14.94 15.58	153 [M + H]^+^, 151 [M-H]^−^ 257 [M + H]^+^, 255 [M-H]^−^ 225 [M + H]^+^ 181 [M + H]^+^	152 256 224 180	12 mm zone of inhibition against MRSA A12;17 mm zone of inhibition against MRSA A1	MW = 152: *o* Hydroxyphenylacetic acid, BE-39907 A2 MW = 256: Phenacein, Albonoursin, N-Isobutyrylpyrrothine, 2,9-Dihydroxyphenazine-1-carboxylic acid
	366 nm	3 bands						
AFD10	254 nm	4 bands	3 yellow bands, 2 brown bands	9.90 12.60 15.61 19.42	211 [M + H]^+^ 137 [M + H]^+^,135 [M-H]^−^ 401 [M + H]^+^, 399 [M-H]^−^ 475 [M + H]^+^, 473 [M-H]^−^	210 136 400 474	17 mm zone of inhibition against MRSA A7; 20 mm zone of inhibition against MRSA A2	MW = 210: 1-Methoxyphenazine, 1-Acetyl-β-carboline. MW = 136: Pyrrole-3-yl-2-propenamide, Anthranilamide, Octalactin C. MW = 400: Daunomycinol, Furaquinocin G, Daidzein-7-*α*-l-rhamnoside, Oxopropaline-E, 2 11-A. MW = 474: *N*-Ethyloxytetracycline, KS-619-1.
	366 nm	1 band						
AFD12	254 nm	3 bands	1 dark yellow band, 1 pink band, 1 green band	3.70 4.36 6.43 12.50 16.06	- 160 [M + H]^+^,158 [M-H]^−^ 110 [M-H]^−^ 205 [M-H]^−^ 137 [M + H]^+^, 135 [M-H]^−^ 211 [M + H]^+^	- 159 111 206 136 210	11 mm zone of inhibition against MRSA A7; 15 mm zone of inhibition against MRSA A5	MW = 159: 4-(Hydroxymethyl)quinolone, Quinazolinamine MW = 136: Pyrrole-3-yl-2-propenamide, Anthranilamide, Octalactin C. MW = 210: Nikkomycin E, (4*R*)-Thiolactomycin, Cyanomycin, 2-Amino-4-hydroxy-3-methyl-4-(3-pyridyl)butanoic acid.
	366 nm	2 bands						
AFD26	254 nm	4 bands	1 brown bands, 1 purple band, 1 light blue band, 1 greenish blue band	.48 6.32 6.38 9.49 10.85 12.23 17.43	1052 [M + H]^+^?? 113 [M + H]^+^, 111 [M-H]^−^ 164 [M + H]^+^, 162 [M-H]^−^ 164 [M + H]^+^, 162 [M-H]^−^ 164 [M + H]^+^, 162 [M-H]^−^ 317 [M + H]^+^ 391 [M + H]^+^	112 163 163 163 316 390	10 mm zone of inhibition against MRSA A1; 15 mm zone of inhibition against MRSA A7	MW = 112: Emimycin, 3,5-Dimethyl-3-oxol-2-on, Uracil, Enimycin. MW = 163: L-β-(3-Hydroxyureido)-alanine, Homoalanosine, β-Phenylethylacetamide, 2-N-Hydroxy-3,4-dihydroisoquinoline-1-one, Streptazone D, 2-Amino-4-hydroxy-pterin, 6-Hydroxy-isatin, Dihydroabikoviromycin, 3-Nitro-1H-indazole, Kitasatodine MW = 316: 17927-D, Boxazomycin A, 1 Isobutyroxymethyl cyclohex-1(6)-ene-2,3,4,5-tetrol-2-isobutyrate. MW = 390: SF-2330, Arizonin C3.
	366 nm	2 bands						

For details, see Supplementary Information (Figure S3-S20)

The total ion chromatogram of strain AFD8 showed various peaks at different retention times, the most prominent of which was observed at 13.42 min, and the molecular mass for this was calculated as 256 Da. The chemical profile of this component was then compared in the database and found the closest match with Phenacein, Isobutyryl pyrrothine and Albonoursin. In the same manner the components appeared at other retention times 9.07 min, 14.94 min and 15.58 min were also compared with the AntiBase (Table 5, Additional file 18: Figure S10, S19, Additional file 15).The total ion chromatogram of strain AFD10 exhibited several peaks at different retention time (*t*_*R*_) ranging from 9.90–19.42 min and the molecular masses calculated for some of the selected peaks were assigned as 210, 136, 400 and 474 Da. The chemical profiles of all of these components were also compared with the compounds in AntiBase and found their close matches e.g. the component with molecular mass 400 Da showed the closest match with Daunomycinol, Furaquinocin G, and Daidzein-7-α-L-rhamnoside (Table 5, Additional file 19: Figure S12, S20, Additional file 20). The crude extract of strain AFD12 also exhibited good LC-MS results as it displayed significant peaks at 4.36–16.06 min, with molecular masses range from 111 to 206 Da. The chemical profiles of all of these components were compared with the database and found their close matches, e.g. the component detected at *t*_*R*_ 4.36 min with molecular mass 159 Da showed a close match with 4-(Hydroxymethyl) quinolone and Quinazolinamine (Table 5, Additional files 21: Figure S14, S20, Additional file 20). While strain AFD14, did not show clear mass based on the HPLC-MS analysis (Additional file 22: Figure S16), it displayed an interesting HPLC-UV/Vis profile (Additional file 12: Figure S15). Finally, the crude extract of strain AFD26 exhibited various peaks at retention times (*t*_*R*_)/molecular masses 6.32 min/112 Da, 6.38 min/163 Da, 9.49 min/163 Da, 10.85 min/163 Da, 12.23 min/316 Da and 17.43 min/390 Da. The chemical profile of all of these components were compared with the data in AntiBase and found the closest matches with reported compounds. For example the component with molecular mass 316 Da showed the close match with Boxazomycin A, 17927-D and Isobutyroxymethyl cyclohex-1(6)-ene-2, 3, 4, 5-tetrol-2-isobutyrate (Table 5, Additional files 23: Figure S18, S20, Additional file 20).

## Discussion

The need for new bioactive metabolites from untapped environments like deserts is mainly stressed due to the drastic consequences and vivacious nature of antibiotic resistance in pathogens. The Cholistan desert of Pakistan is one of those environments which have not been explored yet for the detailed actinobacterial diversity and the search for antimicrobial producing organisms. So, it is anticipated that it may harbor unique and novel species of actinobacteria which can produce new antimicrobial drugs. There is a great need for novel and potential compounds discovery, with their pharmaceutical, industrial or agricultural applications [11]. The actinomycetes are the most authentic sources for new antibiotics because of their varied chemical nature, wide taxonomic scope and environmental distribution. Harsh conditions like dryness, extreme temperatures and alkalinity directed to the isolation of very resistant actinomycetes from untapped environments like deserts, caves etc. Different useful strategies are required to exploit these habitats for the discovery of stress tolerant bacterial species [32].

In the present study 110 actinobacterial strains were isolated from the Cholistan desert. The polyphasic taxonomic characterization including morphological, microscopic, biochemical and physiological characteristics and 16S rRNA gene sequence analysis suggested that these isolates belong to different actinobacterial genera and species. In biochemical characterization it was found that these were able to produce melanin pigment which may be an avoidance strategy to the desiccation and intense radiation, as described by many researchers like Bull and Asenjo [33]. They reported that the actinobacteria living in extreme conditions can be metabolically functional and they may not be considered as dormant. Another very important feature for the identification of actinomycetes especially *Streptomyces* is the determination of utilization of different sugars as a source of carbon. For this purpose these strains were tested for their ability to utilize 8 different sugars (D-fructose, D-glucose, D-galactose, L-arabinose, D-mannitol, sucrose, inositol and mannose). It was found that most of the strains used mainly the glucose as the carbon source, however fructose and sucrose were the next sugars which were used most frequently, these results are comparable with the findings of Aftab et al. [34].

The actinobacterial spores have the capability to germinate in very low available water environments and it enables them to adapt to drought conditions. Our major focus has been on the spore forming actinobacterial diversity, so scanning electron microscopic (SEM) analysis of the aerial spores and substrate mycelium of some of the representative strains showed extensive substrate mycelium and interlocking patterns of the cells, which might be due to the high temperature, high salinity, and radiations effect [10, 35]. A variety of shapes of spores were observed using different magnifications (5 kx, 10 kx, 15 kx, 20 kx, 25 kx, 40 kx and 50 kx), which were arranged in the form of chains and clusters showing various stages of spore development (Fig. 2). The comparison of the spores arrangement and shapes with the descriptions given in Bergey’s manual of systematic bacteriology [29, 30] indicated the major resemblance of these strains with different *Streptomyces* species.

The 16S rRNA gene sequencing and BLAST search via EzTaxon server and subsequent phylogenetic analysis gave the in-depth insights into the actinobacterial diversity in this extreme ecological niche. Mainly the strains belonged to three different families of the order *Actinomycetales*, including the family *Streptomycetaceae*, the family *Pseudonocardiaceae* and the family *Micrococcaceae.* In most of the cases strains shared > 99% genetic similarity with the *Streptomyces* type strains, while 5 of the strains including AFD6, AFD13, AFD23, AFD26 and AFD29 showed < 99% similarity, usually in the range of 97–98% (Fig. 3). This shows that these 5 strains could be different taxon or new *Streptomyces* species; however the complete 16S rRNA gene sequences and other relevant genetic approaches such as DNA-DNA hybridization with the type strain *etc* can further unveil their exact taxonomic status. Okoro et al. [36] reported that majority of the isolates from the soil of Atacama Desert located in south America belonged to the genera *Amycolatopsis*, *Lechevalieria* and *Streptomyces*.

The second major goal in this study was to detect and obtain the actinobacterial strains which exhibit potent activity against MRSA (methicillin resistant *Staphylococcus aureus*). MRSA has been designated as the superbug due to its resistance to almost all the known antibiotic classes. In this scenario activity against MRSA is extremely needed to search the new antimicrobial drug candidates. The actinobacterial strains from this unique ecological environment exhibit remarkable activity against MRSA test strains. Among the 110 strains, in initial screens by agar plug method 30 strains were detected to be active against MRSA. While, further screening through well diffusion method revealed the potent inhibitory activity of these strains against all the MRSA strains. Especially, the strain AFD2 *Streptomyces pseudogriseolus* showed 32 mm zone of inhibition against MRSA. In general, the zone of inhibition in the range of 9–32 mm was observed which is comparable to the findings of Nithya et al. [37], who reported the inhibition of MRSA by desert actinomycetes isolated from Saudi Arabian desert.

The chemical profiling of the crude extracts of these actinobacterial strains showed that they produce compounds belonging to different structural classes, as indicated by colored bands on TLC after staining with anisaldehyde/H_2_SO_4_. For instance, the development of yellow spots indicated the production of actinomycin like compounds, pink spots indicated quinolone like compounds, blue spots were indicative of peptide like fractions, however the peptides are needed to confirm by staining with iodine vapors etc. The greenish spots are usually shown by allylic alcohols, red spots for amines, aldehydes, ketones, carbohydrates and esters (alkyl phthalates) and violet spots for phenols etc. The HPLC/UV chromatograms exhibited a comparative account of the metabolic capability of the isolated actinobacterial strains, as varied number of peaks (up to 7–8 peaks) at different retention times (*t*_*R*_) were observed in the methanolic extracts. This shows that these actinobacterial strains are capable to produce variety of different compounds at the same time with different concentrations. The LC-MS analysis of the strains with prominent activity against MRSA indicated that these actinobacterial strains produce low molecular weight compounds usually < 500 Da. This shows that these active components can be potential antibiotic candidates because the higher molecular weight compounds usually show various cytotoxic effects in the subsequent drug development process. The metabolic profiles of the methanolic extracts of these actinobacterial strains are comparable with those reported by Taddei et al. [38] and Anwar et al. [17], where 5–8 major metabolites has been detected in the extract of one strain.

The comparison of the biological activity and chemical profile of the compounds produced by these strains with those of reported in AntiBase provides deeper insights in to the chemical nature/identity and structural diversity of these active compounds. The AntiBase [28] is a largest and most authentic database with more than 40,000 natural compounds produced by different microorganisms especially by *Streptomyces*. For instance, the strain AFD2 showed the production of a compound which had the close match with the antibiotic Emimycin and Triacsin. In the same manner, the strain AFD4 exhibited the production of different compounds with close matches in database with the antibiotics like 1-Methoxyphenazine and Pyrrole-3-yl-2-propenamide. Similarly, the strain AFD7 exhibited the production of various compounds with close matches with antibiotic compounds such as, Xanthine, Pentenomycin I and Fibrostatin B. The strain AFD8 exhibited the presence of different compounds which had the close matches with the compounds in the AntiBase e.g. *o*-Hydroxyphenylacetic acid and Phenacein. The strain AFD10 showed the production of different compounds which had the close match with the different antibiotic compounds in the database including Anthranilamide and 1-Methoxyphenazine. The strain AFD12 also exhibited the production of different active components which showed the closest matches with 4-(Hydroxymethyl) quinolone and Nikkomycin E. The strain AFD26 exhibited the production of various antibiotic components which had the close matches with Emimycin, Dihydroabikoviromycin and Boxazomycin A (Table 5). The results showed that the diverse spore forming actinobacterial strains are present in this harsh ecological niche and most of them are capable of producing variety of structurally diverse compounds.

## Conclusions

The study revealed that the Cholistan desert in Pakistan harbors an immense and untapped diversity of culturable spore forming actinobacteria with valuable antimicrobial potential against MRSA. The most frequently isolated strains in this study belong to the family *Streptomycetaceae*, and 27 different species of genus *Streptomyces* were identified. The two strains were identified to be the members of different families, such as strain AFD18 *Saccharothrix xinjiangensis* belongs to the family *Pseudonocardiaceae* and the strain AFD22 *Arthrobacter* sp. belongs to the family *Micrococcaceae*. Our findings based on in-depth chemical profiling confirms that most of the strains from this extreme and harsh environment are capable of producing variety of low molecular weight secondary metabolites belonging to different antibiotics structural classes, which should be explored further for new antimicrobial drug candidates.

## Additional files

Additional file 1: Isolation media used for the Actinobacterial Diversity (PDF 279 kb)

Additional file 2: **Table S1.** Primary screening of all the isolated desert actinobacterial strains against MRSA by agar plug method (PDF 201 kb)

Additional file 3: **Table S2.** Microbiological and biochemical characteristics of Cholistan desert actinobacterial strains (PDF 309 kb)

Additional file 4: **Figure S1.** Morphological appearance of selected Cholistan desert actinobacterial strains on GYM agar (A) strain AFD2 *Streptomyces pseudogriseolus* (B) strain AFD3 *Streptomyces canarius* (C) strain AFD6 *Streptomyces fradiae* (D) strain AFD13 *Streptomyces roseofulvus* (E) strain AFD16 *Streptomyces pratensis* (F) strain AFD10 *Streptomyces puniceus (PDF 250 kb)*

Additional file 5: **Figure S2.** Antibacterial activity of the selected actinobacterial strains against MRSA isolates: (A, B) Zone of inhibition in agar plug method, (C, D) zone of inhibition in well method, (E) different bands of crude extracts of actinomycete isolates under UV at 366 nm (F) at 254 nm wavelength. (PDF 232 kb)

Additional file 6: **Figure S3.** HPLC/UV analyses of AFD2 crude extract. HPLC-conditions: Detection wavelength 254 nm; solvent A: H_2_O/0.1% TFA; solvent B: acetonitrile; flow rate: 1.0 mL min^− 1^; 0–30 min, 95–0% A (linear gradient); 30–35 min 0% A; 35–36 min 0–95% A (linear gradient); 36–40 min 95% A. (PDF 224 kb)

Additional file 7 **: Figure S7.** HPLC/UV analyses of AFD7 crude extract. HPLC-conditions: Detection wavelength 254 nm; solvent A: H_2_O/0.1% TFA; solvent B: acetonitrile; flow rate: 1.0 mL min^− 1^; 0–30 min, 95–0% A (linear gradient); 30–35 min 0% A; 35–36 min 0–95% A (linear gradient); 36–40 min 95% A. (PDF 246 kb)

Additional file 8: **Figure S5.** HPLC/UV analyses of AFD4 crude extract. HPLC-conditions: Detection wavelength 254 nm; solvent A: H_2_O/0.1% TFA; solvent B: acetonitrile; flow rate: 1.0 mL min^− 1^; 0–30 min, 95–0% A (linear gradient); 30–35 min 0% A; 35–36 min 0–95% A (linear gradient); 36–40 min 95% A. (PDF 59 kb)

Additional file 9 **: Figure S9.** HPLC/UV analyses of AFD8 crude extract. HPLC-conditions: Detection wavelength 254 nm; solvent A: H_2_O/0.1% TFA; solvent B: acetonitrile; flow rate: 1.0 mL min^− 1^; 0–30 min, 95–0% A (linear gradient); 30–35 min 0% A; 35–36 min 0–95% A (linear gradient); 36–40 min 95% A. (PDF 63 kb)

Additional file 10: **Figure S11.** HPLC/UV analyses of AFD10 crude extract. HPLC-conditions: Detection wavelength 254 nm; solvent A: H_2_O/0.1% TFA; solvent B: acetonitrile; flow rate: 1.0 mL min^− 1^; 0–30 min, 95–0% A (linear gradient); 30–35 min 0% A; 35–36 min 0–95% A (linear gradient); 36–40 min 95% A. (PDF 346 kb)

Additional file 11: **Figure S13.** HPLC/UV analyses of AFD12 crude extract. HPLC-conditions: Detection wavelength 254 nm; solvent A: H_2_O/0.1% TFA; solvent B: acetonitrile; flow rate: 1.0 mL min^− 1^; 0–30 min, 95–0% A (linear gradient); 30–35 min 0% A; 35–36 min 0–95% A (linear gradient); 36–40 min 95% A. (PDF 231 kb)

Additional file 12 **: Figure S15.** HPLC/UV analyses of AFD14 crude extract. HPLC-conditions: Detection wavelength 254 nm; solvent A: H_2_O/0.1% TFA; solvent B: acetonitrile; flow rate: 1.0 mL min^− 1^; 0–30 min, 95–0% A (linear gradient); 30–35 min 0% A; 35–36 min 0–95% A (linear gradient); 36–40 min 95% A. (PDF 242 kb)

Additional file 13 **: Figure S17.** HPLC/UV analyses of AFD26 crude extract. HPLC-conditions: Detection wavelength 254 nm; solvent A: H_2_O/0.1% TFA; solvent B: acetonitrile; flow rate: 1.0 mL min^− 1^; 0–30 min, 95–0% A (linear gradient); 30–35 min 0% A; 35–36 min 0–95% A (linear gradient); 36–40 min 95% A. (PDF 233 kb)

Additional file 14: **Figure S4.** HPLC/MS analyses of AFD2 crude extract. HPLC-conditions: Detection wavelength 254 nm; solvent A: H_2_O/0.1% Formic acid, solvent B: CH_3_CN/0.1% Formic acid; flow rate: 0.5 mL min^− 1^; 0–4 min, 10% B; 4–22 min, 10–100% B; 22–27 min, 100% B; 27–29 min, 100–10% B; 29–30 min, 10% B. (**MW** = **M**olecular **W**eight). (PDF 313 kb)

Additional file 15: **Figure S19.** Chemical structures of the suggested *Streptomyces* metabolites based on HPLC-UV/Vis and LC-MS analyses and by searching in *AntiBase* 2017. (PDF 251 kb)

Additional file 16: **Figure S6.** HPLC/MS analyses of AFD4 crude extract. HPLC-conditions: Detection wavelength 254 nm; solvent A: H_2_O/0.1% Formic acid, solvent B: CH_3_CN/0.1% Formic acid; flow rate: 0.5 mL min^− 1^; 0–4 min, 10% B; 4–22 min, 10–100% B; 22–27 min, 100% B; 27–29 min, 100–10% B; 29–30 min, 10% B. (**MW** = **M**olecular **W**eight). (PDF 421 kb)

Additional file 17 **: Figure S8.** HPLC/MS analyses of AFD7 crude extract. HPLC-conditions: Detection wavelength 254 nm; solvent A: H_2_O/0.1% Formic acid, solvent B: CH_3_CN/0.1% Formic acid; flow rate: 0.5 mL min^− 1^; 0–4 min, 10% B; 4–22 min, 10–100% B; 22–27 min, 100% B; 27–29 min, 100–10% B; 29–30 min, 10% B. (**MW** = **M**olecular **W**eight). (PDF 409 kb)

Additional file 18: **Figure S10.** HPLC/MS analyses of AFD8 crude extract. HPLC-conditions: Detection wavelength 254 nm; solvent A: H_2_O/0.1% Formic acid, solvent B: CH_3_CN/0.1% Formic acid; flow rate: 0.5 mL min^− 1^; 0–4 min, 10% B; 4–22 min, 10–100% B; 22–27 min, 100% B; 27–29 min, 100–10% B; 29–30 min, 10% B. (**MW** = **M**olecular **W**eight) (PDF 385 kb)

Additional file 19: **Figure S12.** HPLC/MS analyses of AFD10 crude extract. HPLC-conditions: Detection wavelength 254 nm; solvent A: H_2_O/0.1% Formic acid, solvent B: CH_3_CN/0.1% Formic acid; flow rate: 0.5 mL min^− 1^; 0–4 min, 10% B; 4–22 min, 10–100% B; 22–27 min, 100% B; 27–29 min, 100–10% B; 29–30 min, 10% B. (**MW** = **M**olecular **W**eight) (PDF 434 kb)

Additional file 20: **Figure S20.** Chemical structures of the suggested *Streptomyces* metabolites based on HPLC-UV/Vis and LC-MS analyses and by searching in *AntiBase* 2017. (PDF 400 kb)

Additional file 21 **: Figure S14.** HPLC/MS analyses of AFD12 crude extract. HPLC-conditions: Detection wavelength 254 nm; solvent A: H_2_O/0.1% Formic acid, solvent B: CH_3_CN/0.1% Formic acid; flow rate: 0.5 mL min^− 1^; 0–4 min, 10% B; 4–22 min, 10–100% B; 22–27 min, 100% B; 27–29 min, 100–10% B; 29–30 min, 10% B. (**MW** = **M**olecular **W**eight) (PDF 431 kb)

Additional file 22 **: Figure S16.** HPLC/MS analyses of AFD14 crude extract. HPLC-conditions: Detection wavelength 254 nm; solvent A: H_2_O/0.1% Formic acid, solvent B: CH_3_CN/0.1% Formic acid; flow rate: 0.5 mL min^− 1^; 0–4 min, 10% B; 4–22 min, 10–100% B; 22–27 min, 100% B; 27–29 min, 100–10% B; 29–30 min, 10% B. No clear mass was detected for this extract, howver it is very intersting based on the HPLC-UV/Vis profile (Fig. S17). (PDF 334 kb)

Additional file 23: **Figure S18.** HPLC/MS analyses of AFD26 crude extract. HPLC-conditions: Detection wavelength 254 nm; solvent A: H_2_O/0.1% Formic acid, solvent B: CH_3_CN/0.1% Formic acid; flow rate: 0.5 mL min^− 1^; 0–4 min, 10% B; 4–22 min, 10–100% B; 22–27 min, 100% B; 27–29 min, 100–10% B; 29–30 min, 10% B. (**MW** = **M**olecular **W**eight). (PDF 410 kb)

## Abbreviations

AMR: Antimicrobial Resistance
BLAST: Basic Local Alignment Search Tool
bp: Base pair
C: Citrate
CLSI: Clinical and Laboratory Standards Institute
DF: D-Fructose
DG: D-Glucose
DGL: D-Galactose
DM: D-Mannitol
DNA: Deoxyribonucleic acid
DNase: Deoxyribonuclease
GIS: Geographic Information System
GYM: Glucose-yeast extract-malt extract
HPLC-UV/Vis: High Performance Liquid Chromatography-Ultraviolet/Visible
I: Inositol
ISP: International *Streptomyces* Project
Km: Kilometers
LA: L-Arabinose
LC-MS: Liquid Chromatography- Mass Spectrometry
M: Mannose
M: Melonate
MEGA6: Molecular Evolutionary Genetics Analysis Version 6
MH: Muller Hinton
mm: Milimeter
MMG: Microbiology and Molecular Genetics
MP: Melanin Production
MRSA: Methicillin Resistant *Staphylococcus aureus*
MW: Molecular Weight
NCBI: National Center for Biotechnology Information
OTUs: Operational Taxonomic Units
PCR: Polymerase Chain Reaction
rRNA: Ribosomal Ribonucleic Acid
S: Sucrose
*S. aureus*: *Staphylococcus aureus*
SE: Secondary Electron
SEM: Scanning Electron Microscopy
TLC: Thin Layer Chromatography
*t*_*R*_: Retention Time
UV: Ultraviolet
