# Supplementary material for: Spore forming Actinobacterial diversity of Cholistan Desert Pakistan: Polyphasic taxonomy, antimicrobial potential and chemical profiling
Source: BMC Microbiol. 2019 Feb 22;19:49. doi: 10.1186/s12866-019-1414-x (PMC6387500; doi:10.1186/s12866-019-1414-x)
Supplement: Supplementary file 1 — Isolation media used for the Actinobacterial Diversity (PDF 279 kb) [file 12866_2019_1414_MOESM1_ESM.pdf]

Isolation media used for the Actinobacterial Diversity

Two different media were used. The recipes and cultural conditions used as followed:

1. **Glycerol-casein-KNO_3 Agar**

Ingredients	Quantity
Glycerol	10.0g
KNO_3	2.0g
Casein	0.3g
NaCl	2.0g
K_2HPO_4	2.0g
$MgSO_4.7H_2O$	0.05g
$CaCO_3$	0.02g
$FeSO_4.7H_2O$	0.01g
Agar	18.0g

Distilled water: 1000 liter, pH: 7.8

Sterilize by autoclaving at 15 lbs pressure (121°C) for 15 minutes. Agitate constantly while pouring into sterile Petri plates to obtain a uniform suspension. Incubation is done at 28°C for 5 to 7 days.

2. **Actinomycete Isolation Agar**

Ingredients	Quantity
Glycerol	5.0g
Sodium propionate	4.0g
Sodium caseinate	2.0g
Asparagine	0.1g
K_2HPO_4	0.5g
$MgSO_4.7H_2O$	0.1g
$FeSO_4.7H_2O$	0.001
Agar	18.0g

Distilled water: 1000 liter, pH: 7.8

Sterilize by autoclaving at 15 lbs pressure (121°C) for 15 minutes. Agitate constantly while pouring into sterile Petri plates to obtain a uniform suspension. Incubation is done at 28°C for 5 to 7 days.
