# Supplementary material for: Spore forming Actinobacterial diversity of Cholistan Desert Pakistan: Polyphasic taxonomy, antimicrobial potential and chemical profiling
Source: BMC Microbiol. 2019 Feb 22;19:49. doi: 10.1186/s12866-019-1414-x (PMC6387500; doi:10.1186/s12866-019-1414-x)
Supplement: Supplementary file 2 — Table S1. Primary screening of all the isolated desert actinobacterial strains against MRSA by agar plug method (PDF 201 kb) [file 12866_2019_1414_MOESM2_ESM.pdf]

Table S1 Primary screening of all the isolated desert actinobacterial strains against MRSA by agar plug method

Actinomycetes strains	*Zone of inhibition against MRSA	Actinomycetes strains	*Zone of inhibition against MRSA	Actinomycetes strains	*Zone of inhibition against MRSA	Actinomycetes strains	*Zone of inhibition against MRSA
AD1	6	**AD29**	**17**	**AD57**	**20**	**AD85**	**14**
AD2	10	AD30	10	AD58	9	AD86	9
**AD3**	**18**	AD31	11	**AD59**	**17**	AD87	9
AD4	9	AD32	-	AD60	10	AD88	-
AD5	-	AD33	8	**AD61**	**15**	**AD89**	**16**
**AD6**	**17**	**AD34**	**17**	AD62	-	AD90	8
AD7	7	AD35	8	**AD63**	**17**	AD91	8
AD8	7	AD36	9	AD64	9	AD92	9
AD9	5	**AD37**	**18**	AD65	8	AD93	10
**AD10**	**19**	**AD38**	**18**	AD66	5	AD94	-
AD11	18	AD39	10	AD67	9	AD95	-
AD12	-	AD40	-	**AD68**	**18**	**AD96**	**14**
AD13	9	AD41	9	**AD69**	**19**	AD97	9
AD14	6	**AD42**	**15**	AD70	-	AD98	5
**AD15**	**18**	AD43	9	AD71	-	AD99	6
AD16	10	AD44	8	AD72	-	**AD100**	**14**
AD17	9	AD45	8	**AD73**	**21**	AD101	-
AD18	9	AD46	-	AD74	9	AD102	-
AD19	-	AD47	-	AD75	9	AD103	9
**AD20**	**15**	**AD48**	**15**	**AD76**	**16**	**AD104**	**15**
**AD21**	**17**	AD49	7	AD77	10	AD105	8
AD22	8	AD50	6	AD78	-	AD106	7
**AD23**	**23**	AD51	6	AD79	-	AD107	18
AD24	10	AD52	-	**AD80**	**17**	**AD108**	**7**
AD25	7	AD53	9	**AD81**	**15**	AD109	8
AD26	7	**AD54**	**17**	AD82	9	AD110	9
AD27	-	AD55	-	AD83	9		
AD28	-	AD56	9	AD84	-		

*zone of inhibition measured in mm; active strains are boldfaced in the whole collection
