# Supplementary material for: Spore forming Actinobacterial diversity of Cholistan Desert Pakistan: Polyphasic taxonomy, antimicrobial potential and chemical profiling
Source: BMC Microbiol. 2019 Feb 22;19:49. doi: 10.1186/s12866-019-1414-x (PMC6387500; doi:10.1186/s12866-019-1414-x)
Supplement: Supplementary file 3 — Table S2. Microbiological and biochemical characteristics of Cholistan desert actinobacterial strains (PDF 309 kb) [file 12866_2019_1414_MOESM3_ESM.pdf]

Table S2 Microbiological and biochemical characteristics of Cholistan desert actinobacterial strains

Strains	Microbiological Characteristics (Colony characteristics)					Biochemical Characteristics (Production of melanin and Utilization of different sugars as sole source of carbon)								
	**Growth Pattern**	**Diffused pigments**	**Consistency**	**Size**	**Shape**	**POM**	**DG**	**DF**	**LA**	**DM**	**S**	**I**	**DGL**	**M**
AFD31	Good	No	Hard embedded	2mm	Round	+	+	+	+	+	+	+	+	+
AFD32	Moderate	No	Hard embedded	2mm	Round	-	+	+	+	+	+	+	+	+
AFD33	Minor	No	-	-	-	-	+	-	-	-	-	-	-	-
AFD34	Minor	No	-	2mm	-	-	+	-	-	-	+	-	-	-
AFD35	Good	No	-	-	-	-	+	-	-	+	-	-	-	+
AFD36	Good	No	-	-	-	-	+	-	+	-	-	-	+	+
AFD37	Minor	No	-	2mm	Round	-	+	-	-	-	-	-	-	+
AFD38	Minor	No	-	-	-	-	+	+	+	-	-	+	-	-
AFD39	Minor	No	-	-	-	+	+	+	+	+	+	-	+	-
AFD40	Good	No	Soft spores	2mm	-	-	+	+	-	-	+	+	-	+
AFD41	Minor	No	-	-	-	+	+	-	-	+	-	+	+	+
AFD42	Minor	No	-	-	-	-	+	-	-	-	-	-	-	+
AFD43	Light	No	-	-	-	-	+	+	+	+	-	-	-	+
AFD44	Light	No	-	-	-	-	+	-	+	-	-	-	-	+
AFD45	Light	No	-	-	-	-	+	-	-	-	-	-	+	+
AFD46	Minor	No	-	-	-	-	+	-	+	-	-	-	-	+
AFD47	Light	No	-	-	-	-	+	-	-	-	-	-	-	+
AFD48	Light	No	Soft spores	-	Round	-	+	-	-	-	-	-	+	+
AFD49	Minor	No	-	-	-	-	+	+	-	-	+	-	-	+
AFD50	Minor	No	-	-	-	-	-	+	-	-	-	-	-	+
AFD51	Light	Yellow	Soft spores	-	Round	+	+	-	-	-	-	+	-	-
AFD52	Minor	No		-	-	+	+	+	-	+	+	+	+	+
AFD53	Minor	No	-	-	-	-	+	-	-	-	-	-	-	-
AFD54	Minor	No	-	-	-	-	+	-	-	-	-	-	-	+
AFD55	Minor	No	-	-	-	-	+	-	+	-	-			+
AFD56	Moderate	No	Hard embedded	-	-	+	+	-	-	+	-	+	-	+
AFD57	Moderate	Brown	-	2mm	Round	-	+	+	-	+	-	-	+	+
AFD58	Moderate	No	-	2mm	-	-	+	+	+	+	-	-	+	+
AFD59	Minor	No	-	-	Round	-	-	-	-	-	-	-	-	-
AFD60	Minor	No	-	2mm	-	-	+	+	-	+	-		+	+
AFD61	Minor	No	-	-	-	-	+	+	-	+	+		+	+
AFD62	Good	No	-	2mm	-	-	+	+	-	+	-	+	+	+
AFD63	Good	No	-	2mm	-	+	+	-	+	-	+	+	+	-
AFD64	Minor	No	-	-	-	-	+	-	-	-	-	-	-	-
AFD65	Minor	No	-	-	-	-	+	-	+	-	-	+	-	-
AFD66	Light	No	-	-	-	-	+	-	-	-	-	-	+	+
AFD67	Light	No	Soft	-	-	-	+	-	-	-	-	-	-	-
AFD68	-	No	-	-	-	-	-	-	-	-	-	-	-	-
AFD69	Minor	No	-	-	-	-	-	-	-	-	-	-	-	-
AFD70	Minor	No	Soft	-	-	-	+	-	-	-	-	-	+	-
AFD71	Moderate	No	Soft	-	-	-	+	-	-	+	-	-	-	+
AFD72	Good	No	-	2mm	Round	-	+	-	+	+	-	-	+	+
AFD73	Good	No	-	2mm	Round	+	+	-	+	-	-	+	-	+
AFD74	Good	No	-	-	-	-	+	+	-	-	-	-	+	+
AFD75	Good	No	Soft	-	-	-	+	+	+	-	-	-	-	+
AFD76	-	-	-	-	-	-	-	-	-	-	-	-	-	-
AFD77	Minor	No	-	-	-	+	+	-	-	-	-	-	-	-
AFD78	Minor	No	-	-	-	-	+	-	-	-	-	-	-	-
AFD79	Minor	No	-	-	-	-	+	-	-	-	-	-	-	-

AFD80	Minor	No	-	-	-	-	+	-	-	-	-	-	-	-
AFD81	Minor	No	-	2mm	Round	-	+	-	-	-	-	-	-	-
AFD82	Minor	No	-	2mm	-	-	+	-	-	-	-	-	-	-
AFD83	Minor	No	-		-	-	+	-	-	-	-	-	-	-
AFD84	Minor	No	-		-	-	+	-	-	-	-	-	-	-
AFD85	-	-	-	-	-	-	-	-	-	-	-	-	-	-
AFD86	Moderate	No	Soft	Pinpoint	-	+	+	+			-	-	+	-
AFD87	Moderate	No	-	Pinpoint	Round	+	+	+	-	+	-	-	+	-
AFD88	Moderate	No	-	-	-	-	+	+	-	+	-	-	+	-
AFD89	Good	No	-	-	-	+	-	-	-	-	-	-	+	-
AFD90	Good	No	-	-	-	+	+	+	-	+	-	-	+	-
AFD91	-	-	-	-	-	-	-	-	-	-	-	-	-	-
AFD92	Good	No	Soft		-	+	+	+	-	+	-	-	+	-
AFD93	Light	No	Soft	Pinpoint	Round	-	+	-	-	-	-	-	-	-
AFD94	Minor	No	-	-	-	-	+	-	-	-	-	-	-	-
AFD95	Minor	No	-	-	-	-	+	-	-	-	-	-	-	-
AFD96	Moderate	No	-	-	-	+	-	+	-	+	-	-	+	-
AFD97	-	-	-	-	-	-	-	-	-	-	-	-	-	-
AFD98	Moderate	No	-	-	-	+	+	-	+	+	-	-	+	-
AFD99	Moderate	No	-	-	-	+	+	+	-	-	-	-	+	-
AFD100	Moderate	No	Soft	-	-	-	+	+	-	-	-	-	+	-
AFD101	Moderate	No	Soft	-	-	+	+	+	-	+	-	-	+	-
AFD102	Good	No	Soft	Pinpoint	Round	+	+	+	-	+	-	-	+	-
AFD103	-	-	-	-	-	-	-	-	-	-	-	-	-	-
AFD104	Good	No	-	Pinpoint	-	+	+	+	-	+	-	-	+	-
AFD105	Good	No	-	-	-	+	+	+	-	+	-	-	+	-
AFD106	Good	No	-	-	-	+	+	+	-	+	-	-	-	-
AFD107	Minor	No	-	-	-	-	-	-	-	-	-	-	-	-
AFD108	Good	No	-	-	-	+	-	-	-	-	-	-	-	-
AFD109	Minor	No	-	-	-	-	+	-	-	-	-	-		-
AFD110	Minor	-	-	-	-	-	+	-	-	-	-	-	-	-

Key: POM= production of melanin, DG=D-glucose, DF=D-fructose, LA=L-arabinose, DM=D-mannitol, S=Sucrose, I=Inositol, DGL=D-galactose, M=Mannose, (+) = positive result, (-) = negative result
