# Supplementary material for: Spore forming Actinobacterial diversity of Cholistan Desert Pakistan: Polyphasic taxonomy, antimicrobial potential and chemical profiling
Source: BMC Microbiol. 2019 Feb 22;19:49. doi: 10.1186/s12866-019-1414-x (PMC6387500; doi:10.1186/s12866-019-1414-x)
Supplement: Supplementary file 4 — Figure S1. Morphological appearance of selected Cholistan desert actinobacterial strains on GYM agar (A) strain AFD2 Streptomyces pseudogriseolus (B) strain AFD3 Streptomyces canarius (C) strain AFD6 Streptomyces fradiae (D) strain AFD13 Streptomyces roseofulvus (E) strain AFD16 Streptomyces pratensis (F) strain AFD10 Streptomyces puniceus (PDF 250 kb) [file 12866_2019_1414_MOESM4_ESM.pdf]

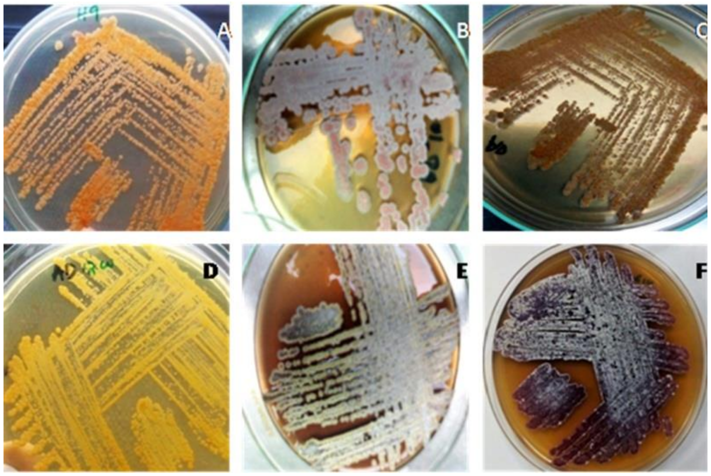

Figure S1: Morphological appearance of selected Cholistan desert actinobacterial strains on GYM agar (A) strain AFD2 *Streptomyces pseudogriseolus* (B) strain AFD3 *Streptomyces canarius* (C) strain AFD6 *Streptomyces fradiae* (D) strain AFD13 *Streptomyces roseofulvus* (E) strain AFD16 *Streptomyces pratensis* (F) strain AFD10 *Streptomyces puniceus*
