# Supplementary material for: Spore forming Actinobacterial diversity of Cholistan Desert Pakistan: Polyphasic taxonomy, antimicrobial potential and chemical profiling
Source: BMC Microbiol. 2019 Feb 22;19:49. doi: 10.1186/s12866-019-1414-x (PMC6387500; doi:10.1186/s12866-019-1414-x)
Supplement: Supplementary file 5 — Figure S2. Antibacterial activity of the selected actinobacterial strains against MRSA isolates: (A, B) Zone of inhibition in agar plug method, (C, D) zone of inhibition in well method, (E) different bands of crude extracts of actinomycete isolates under UV at 366 nm (F) at 254 nm wavelength. (PDF 232 kb) [file 12866_2019_1414_MOESM5_ESM.pdf]

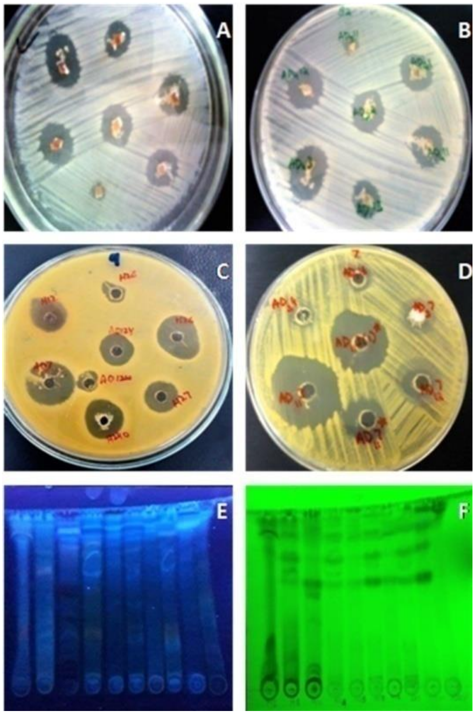

Figure S2: Antibacterial activity of the selected actinobacterial strains against MRSA isolates: (A, B) Zone of inhibition in agar plug method, (C, D) zone of inhibition in well method, (E) different bands of crude extracts of actinomycete isolates under UV at 366nm (F) at 254nm wavelength.
