# Supplementary material for: Spore forming Actinobacterial diversity of Cholistan Desert Pakistan: Polyphasic taxonomy, antimicrobial potential and chemical profiling
Source: BMC Microbiol. 2019 Feb 22;19:49. doi: 10.1186/s12866-019-1414-x (PMC6387500; doi:10.1186/s12866-019-1414-x)
Supplement: Supplementary file 11 — Figure S13. HPLC/UV analyses of AFD12 crude extract. HPLC-conditions: Detection wavelength 254 nm; solvent A: H2O/0.1% TFA; solvent B: acetonitrile; flow rate: 1.0 mL min− 1; 0–30 min, 95–0% A (linear gradient); 30–35 min 0% A; 35–36 min 0–95% A (linear gradient); 36–40 min 95% A. (PDF 231 kb) [file 12866_2019_1414_MOESM11_ESM.pdf]

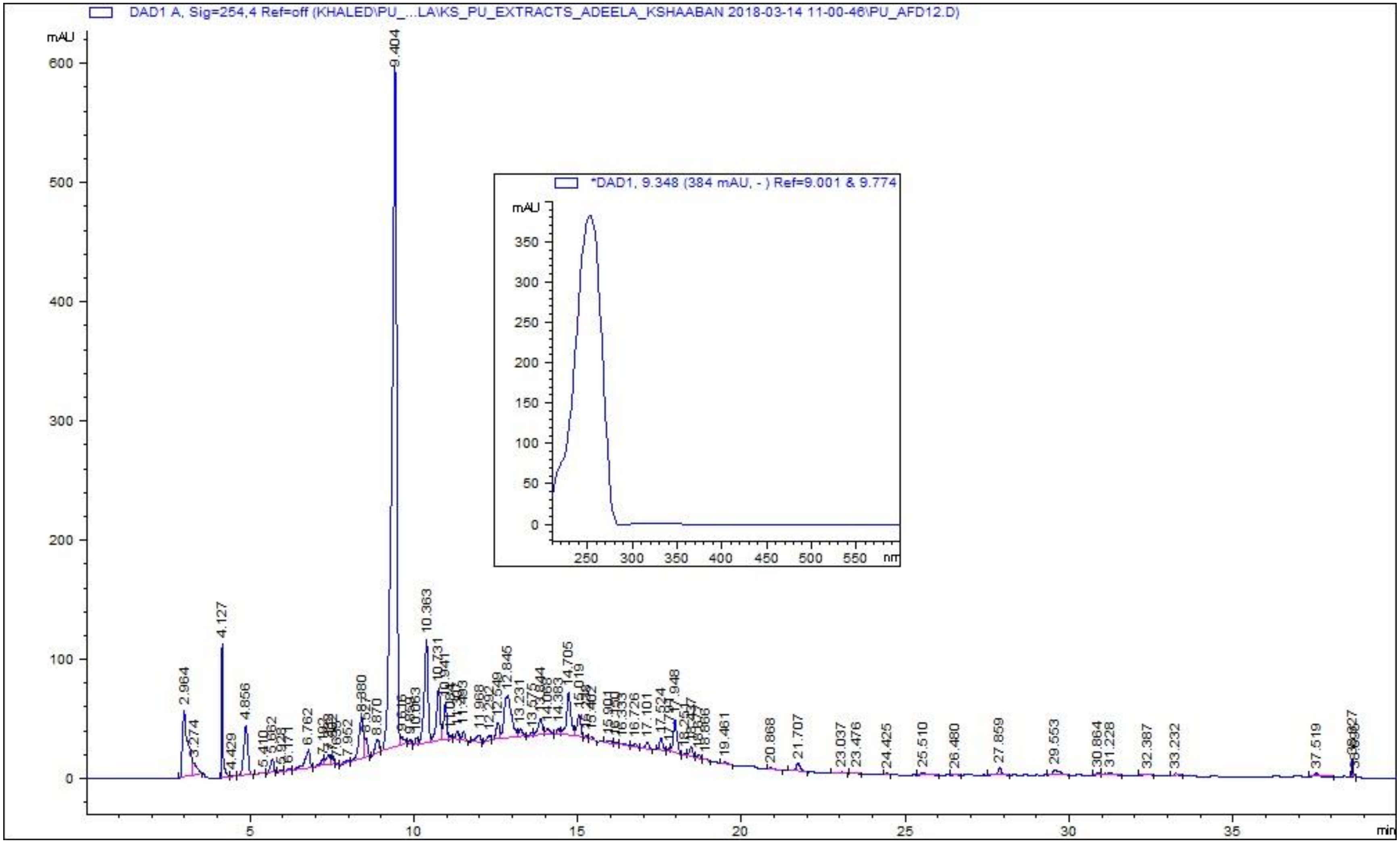

Figure S13. HPLC/UV analyses of AFD12 crude extract. HPLC-conditions: Detection wavelength 254 nm; solvent A: H_2O/0.1% TFA; solvent B: acetonitrile; flow rate: 1.0 mL min^{-1}; 0-30 min, 95-0% A (linear gradient); 30-35 min 0% A; 35-36 min 0-95% A (linear gradient); 36-40 min 95% A.
