# Supplementary material for: Spore forming Actinobacterial diversity of Cholistan Desert Pakistan: Polyphasic taxonomy, antimicrobial potential and chemical profiling
Source: BMC Microbiol. 2019 Feb 22;19:49. doi: 10.1186/s12866-019-1414-x (PMC6387500; doi:10.1186/s12866-019-1414-x)
Supplement: Supplementary file 15 — Figure S19. Chemical structures of the suggested Streptomyces metabolites based on HPLC-UV/Vis and LC-MS analyses and by searching in AntiBase 2017. (PDF 251 kb) [file 12866_2019_1414_MOESM15_ESM.pdf]

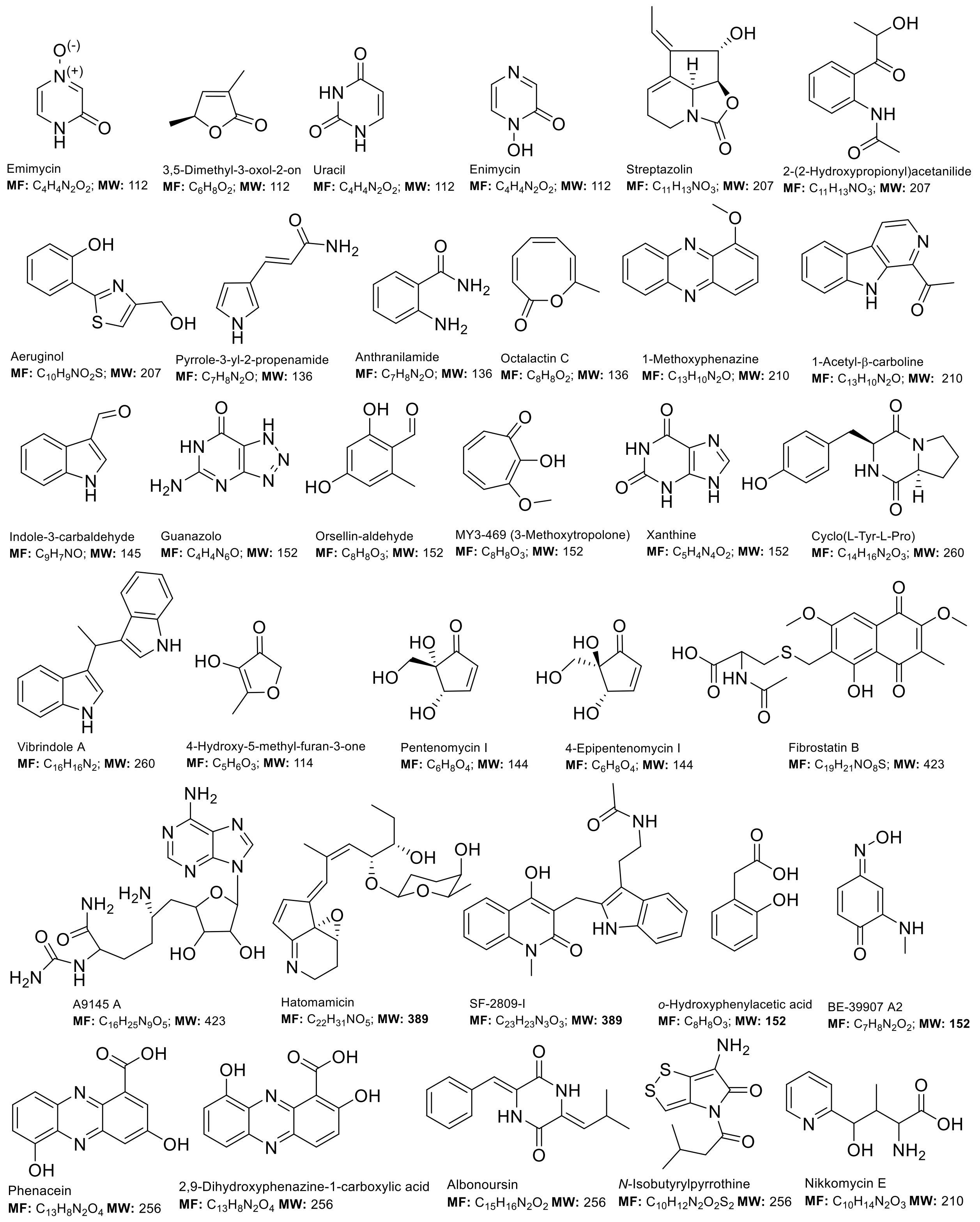

Figure S19: Chemical structures of the suggested *Streptomyces* metabolites based on HPLC-UV/Vis and LC-MS analyses and by searching in *AntiBase* 2017.
