# Supplementary material for: Spore forming Actinobacterial diversity of Cholistan Desert Pakistan: Polyphasic taxonomy, antimicrobial potential and chemical profiling
Source: BMC Microbiol. 2019 Feb 22;19:49. doi: 10.1186/s12866-019-1414-x (PMC6387500; doi:10.1186/s12866-019-1414-x)
Supplement: Supplementary file 17 — : Figure S8. HPLC/MS analyses of AFD7 crude extract. HPLC-conditions: Detection wavelength 254 nm; solvent A: H2O/0.1% Formic acid, solvent B: CH3CN/0.1% Formic acid; flow rate: 0.5 mL min− 1; 0–4 min, 10% B; 4–22 min, 10–100% B; 22–27 min, 100% B; 27–29 min, 100–10% B; 29–30 min, 10% B. (MW = Molecular Weight). (PDF 409 kb) [file 12866_2019_1414_MOESM17_ESM.pdf]

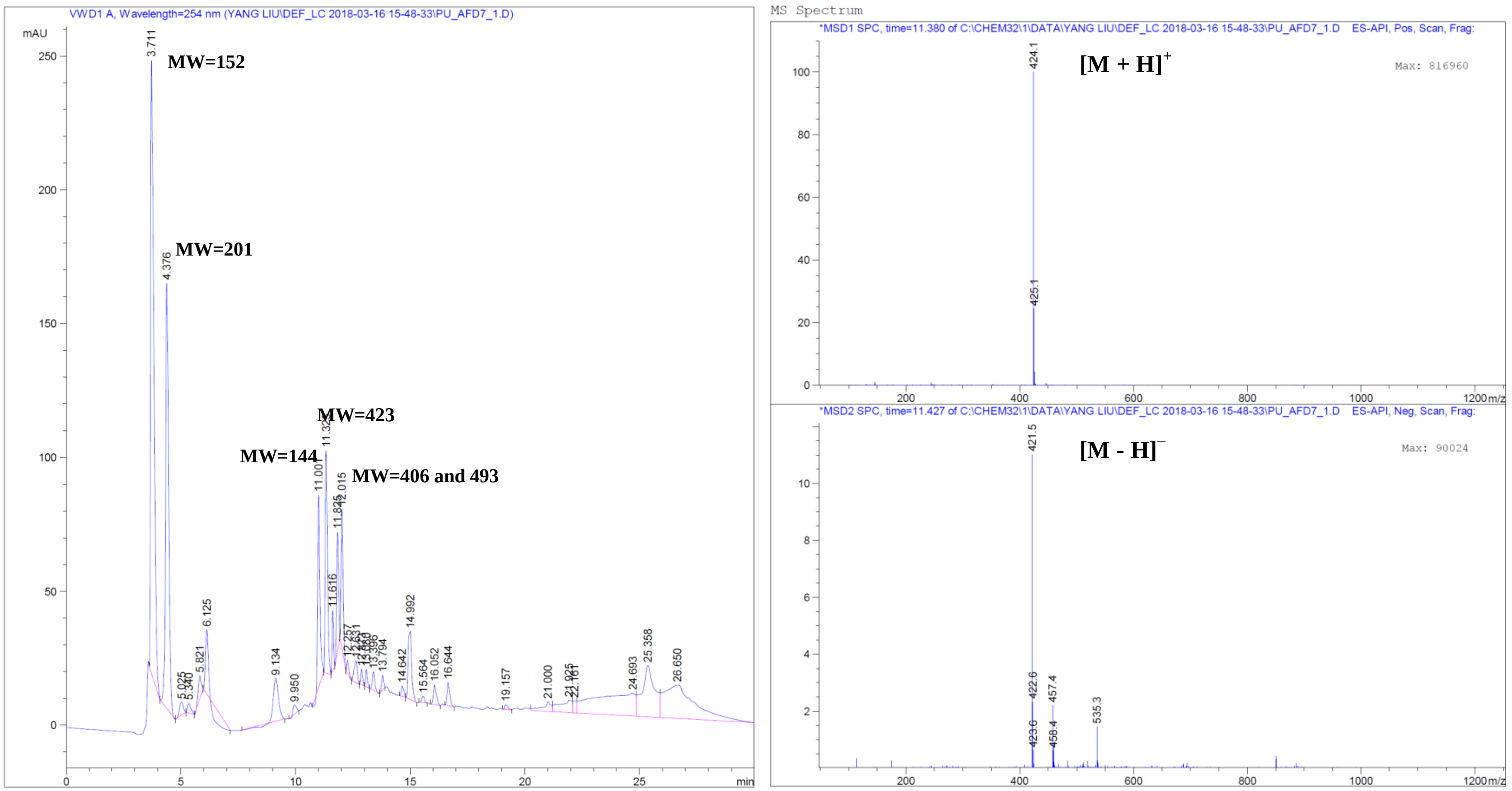


**Figure S8.** HPLC/MS analyses of AFD7 crude extract. HPLC-conditions: Detection wavelength 254 nm; solvent A: $H_2O$/0.1% Formic acid, solvent B: $CH_3CN$/0.1% Formic acid; flow rate: 0.5 mL $min^{-1}$; 0-4 min, 10% B; 4-22 min, 10-100% B; 22-27 min, 100% B; 27-29 min, 100%-10% B; 29-30 min, 10 % B. (**MW** = **M**olecular **W**eight)
